# Supplementary material for: Extensive off-fault damage around the 2023 Kahramanmaraş earthquake surface ruptures
Source: Nat Commun. 2025 Feb 3;16:1286. doi: 10.1038/s41467-025-56466-w (PMC11791046; doi:10.1038/s41467-025-56466-w)
Supplement: Supplementary file 1 — Supplementary Information [file 41467_2025_56466_MOESM1_ESM.pdf]

Supplementary Information for

Extensive off-fault damage around the 2023 Kahramanmaraş earthquake surface ruptures

Jihong Liu[1], Sigurjón Jónsson[1,*], Xing Li[1], Wenqian Yao[2,3], and Yann Klinger[2]

[1]King Abdullah University of Science and Technology (KAUST), Thuwal, 23955, Saudi Arabia
[2]Université Paris Cité, Institut de Physique du Globe de Paris, CNRS, F-75005 Paris, France
[3]Institute of Surface-Earth System Science, School of Earth System Science, Tianjin University, Tianjin, China
[*]Corresponding author: Sigurjón Jónsson (sigurjon.jonsson@kaust.edu.sa)

This Supplementary Information section includes:

Supplementary Figs. **1-30**

Supplementary Tables **1-2**

Supplementary Discussion **1**: Do geodetic slip inversions using simple homogeneous elastic half-space models significantly under- or overestimate shallow slip deficit?

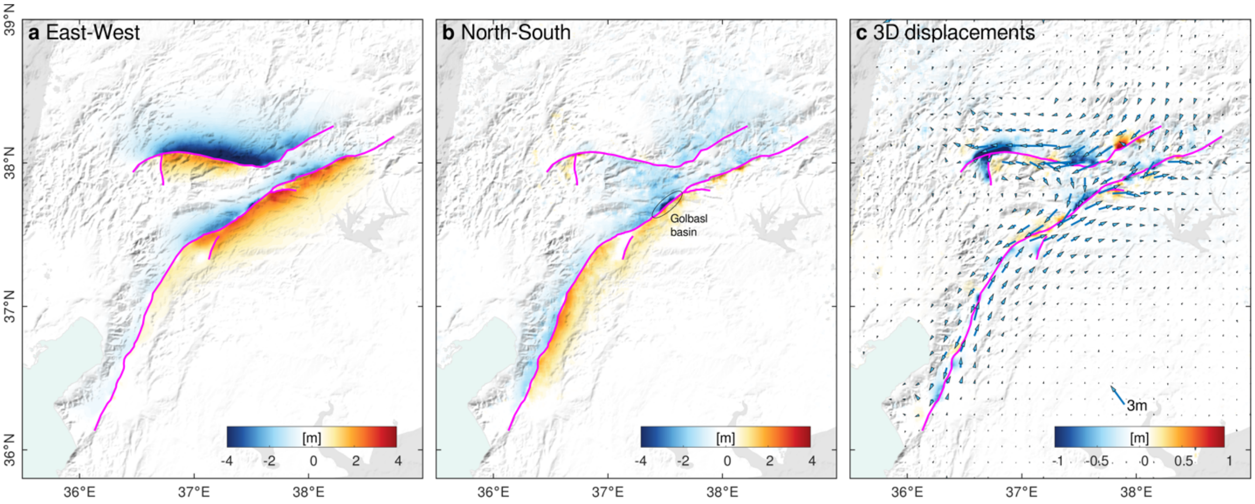

Supplementary Fig. 1 Coseismic three-dimensional surface displacements of the 2023 Kahramanmaraş (Türkiye) earthquakes calculated based on the strain model and variance component estimation (SM-VCE) method[1]. In **c**, arrows show downsampled horizontal displacements and the background map shows vertical displacements. Magenta lines are the surface ruptures mapped from SAR-based observations. The map background shows the elevation of the study region derived from the Shuttle Radar Topography Mission (SRTM) 3-arc seconds data[2].

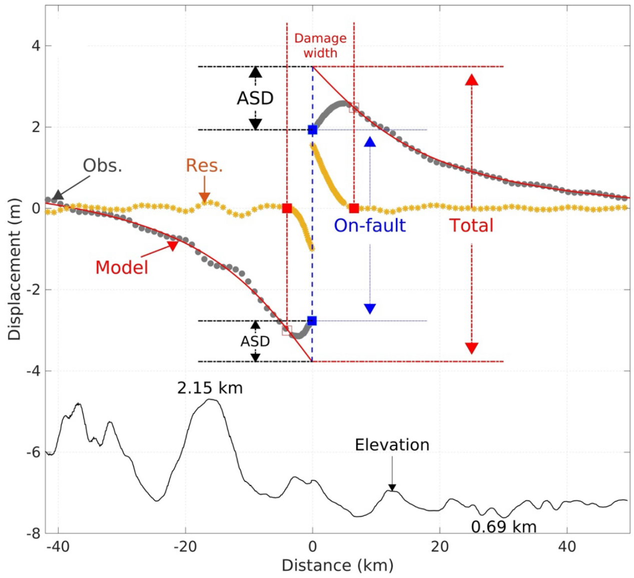

Supplementary Fig. 2. Same as Fig. 2d in the main text, but with additional elevation profile, suggesting that the ~5 km length scale of residuals (yellow) at around -20 km could be attributed to topography-related signals. This is reasonable since the accuracy of the pixel-offset tracking (POT) displacement from non-orthorectified SAR images is generally affected by the local topography[3].

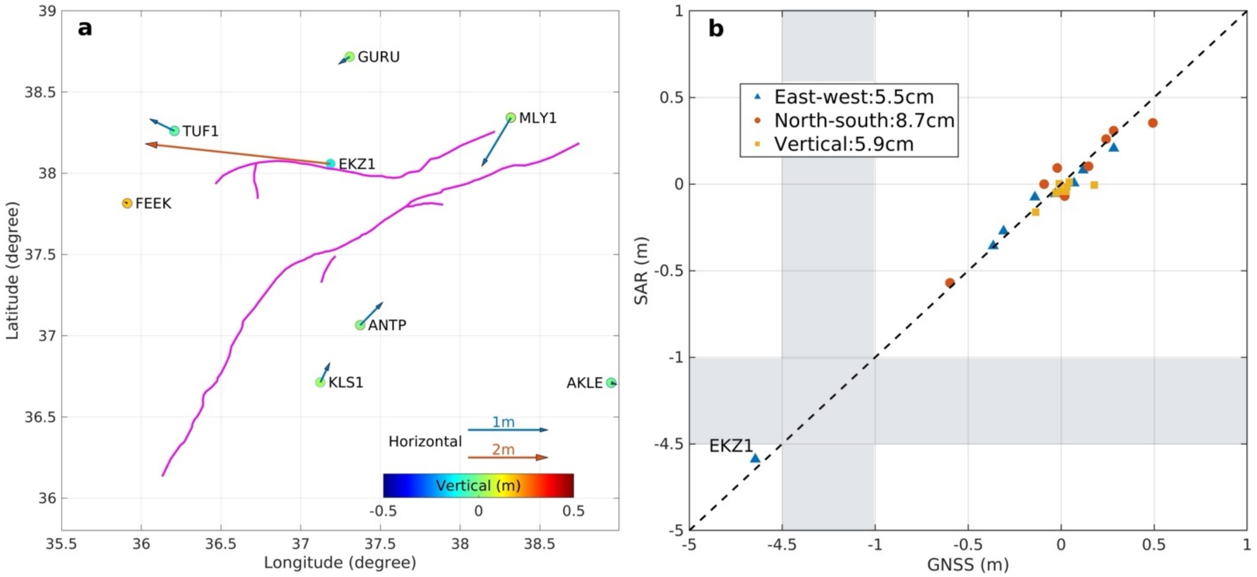

Supplementary Fig. 3 Comparison between global navigation satellite system (GNSS) and SAR-derived coseismic three-dimensional (3D) surface displacements, i.e., east, north, and vertical displacements. **a** GNSS 3D displacements obtained from the Nevada Geodetic Laboratory with arrows and colored circles representing horizontal and vertical displacements, respectively. The GNSS station name is labeled beside each station. Magenta lines are the surface fault ruptures. **b** Comparison between GNSS and SAR-derived 3D displacements. For better display, the interval [-4.5, -1] of x-/y-axis is compressed (in gray). The numbers in the legend represent the root mean square error (RMSE) of the three components, which are calculated as the standard deviation of the difference between the GNSS and SAR-derived displacements.

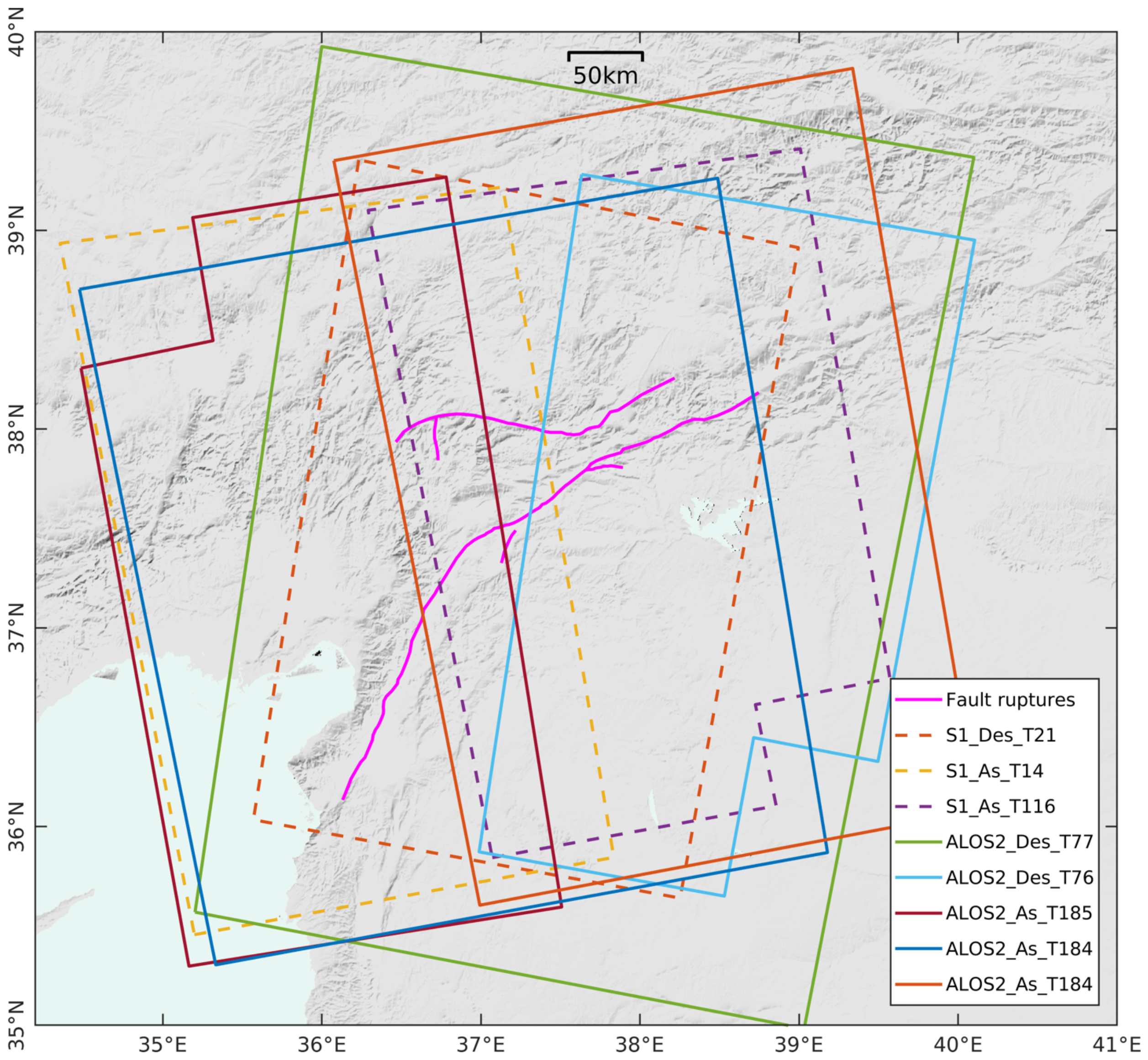

Supplementary Fig. 4 The footprint of the satellite synthetic aperture radar (SAR) images used in this study. S1, Sentinel-1; ALOS-2, Advanced Land Observing Satellite-2; Des, Descending; As, Ascending; T, Track. The map background shows the elevation of the study region derived from the Shuttle Radar Topography Mission (SRTM) 3-arc seconds data[2]. Magenta lines are the surface ruptures mapped from SAR-based observations.

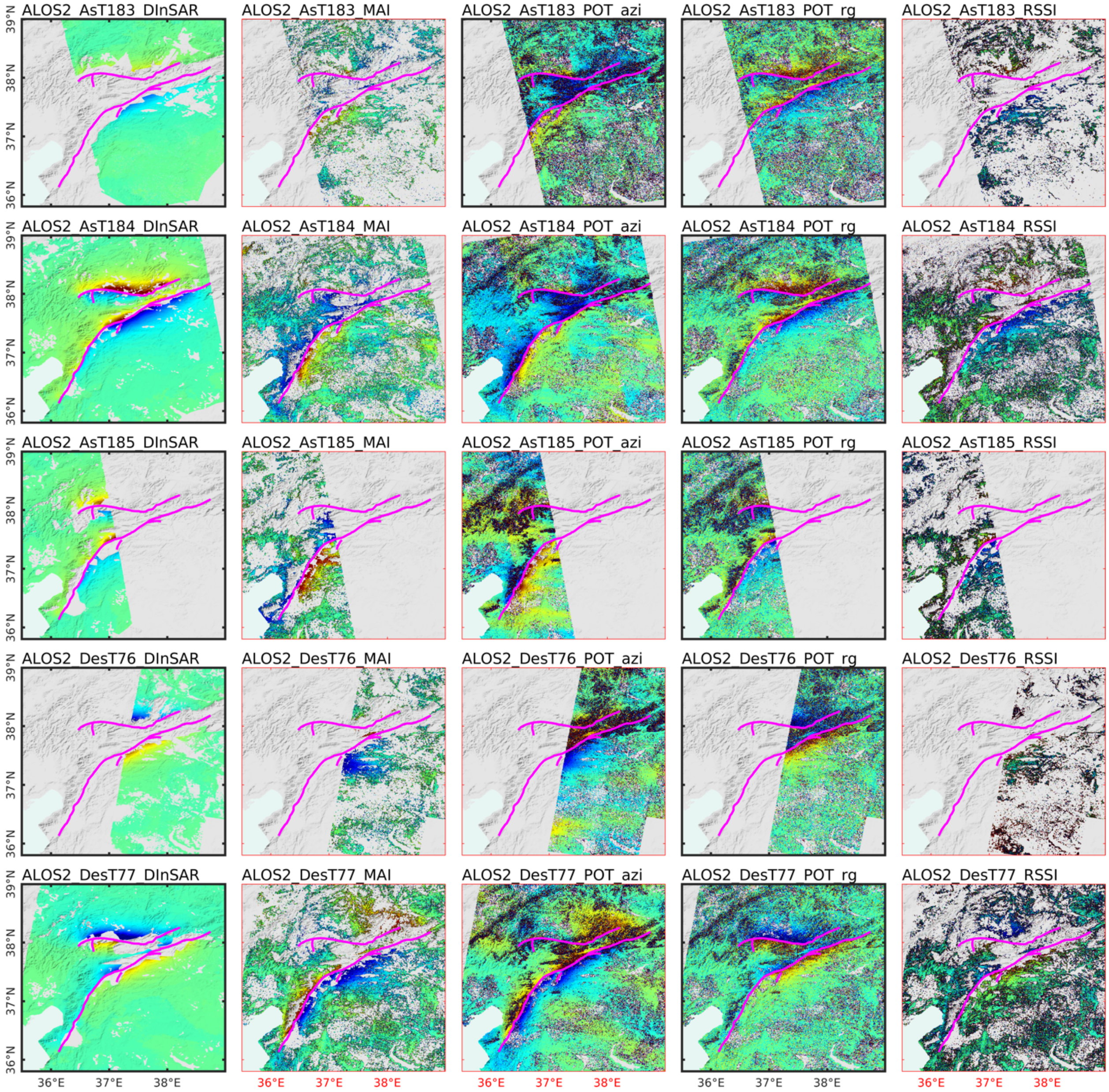

ALOS2_AsT183_DInSAR
ALOS2_AsT183_MAI
ALOS2_AsT183_POT_azi
ALOS2_AsT183_POT_rg
ALOS2_AsT183_RSSI
ALOS2_AsT184_DInSAR
ALOS2_AsT184_MAI
ALOS2_AsT184_POT_azi
ALOS2_AsT184_POT_rg
ALOS2_AsT184_RSSI
ALOS2_AsT185_DInSAR
ALOS2_AsT185_MAI
ALOS2_AsT185_POT_azi
ALOS2_AsT185_POT_rg
ALOS2_AsT185_RSSI
ALOS2_DesT76_DInSAR
ALOS2_DesT76_MAI
ALOS2_DesT76_POT_azi
ALOS2_DesT76_POT_rg
ALOS2_DesT76_RSSI
ALOS2_DesT77_DInSAR
ALOS2_DesT77_MAI
ALOS2_DesT77_POT_azi
ALOS2_DesT77_POT_rg
ALOS2_DesT77_RSSI
39°N
38°N
37°N
36°N
36°E
37°E
38°E

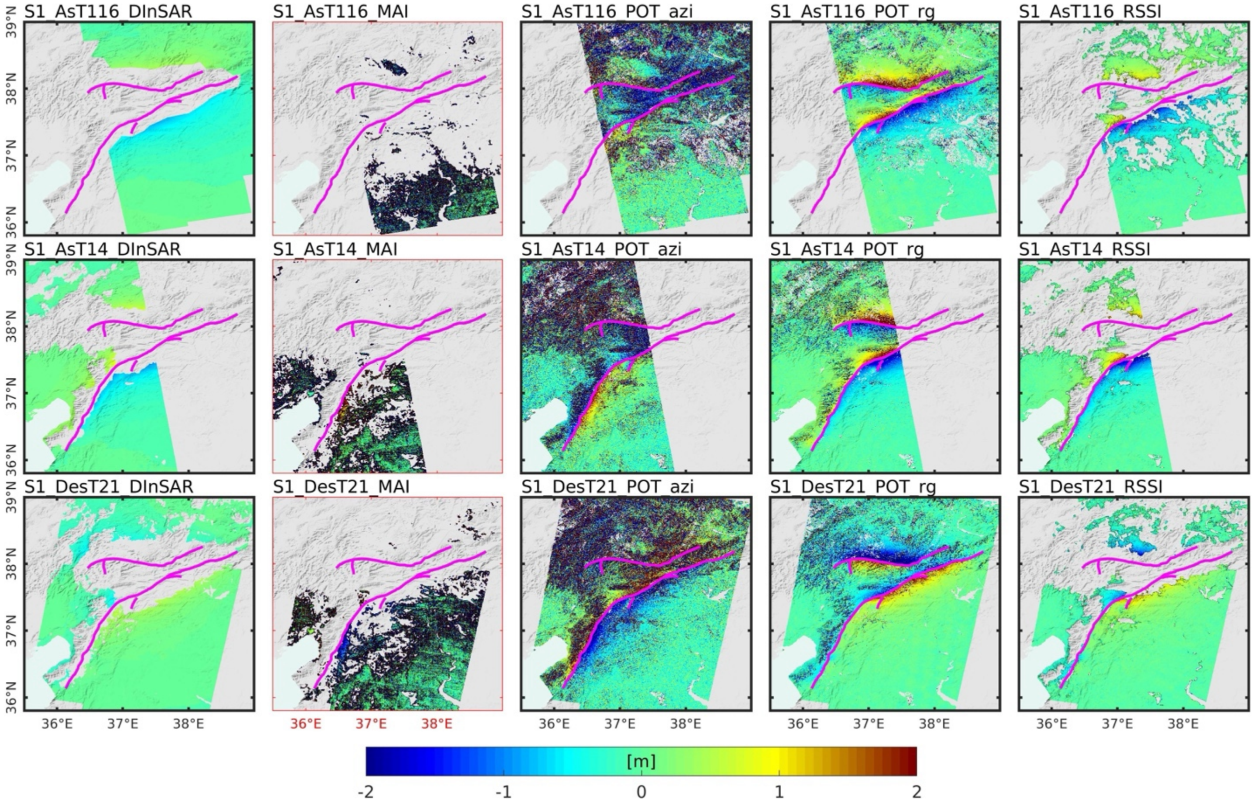

Supplementary Fig. 5 Displacement observations from Sentinel-1 (S1) and ALOS-2 SAR images from interferometric SAR (InSAR), multiple aperture interferometry (MAI), pixel-offset tracking (POT), and range spectrum split interferometry (RSSI). azi, azimuth direction; rg, range direction. Observations framed with a thin red line were excluded when estimating three-dimensional displacements due to decorrelation (i.e., the S1 MAI and ALOS-2 RSSI observations) or ionospheric delay effects in observations along the azimuth direction (i.e., the ALOS-2 MAI and POT_az observations). The map background shows the elevation of the study region derived from the Shuttle Radar Topography Mission (SRTM) 3-arc seconds data[2]. Magenta lines are the surface ruptures mapped from SAR-based observations.

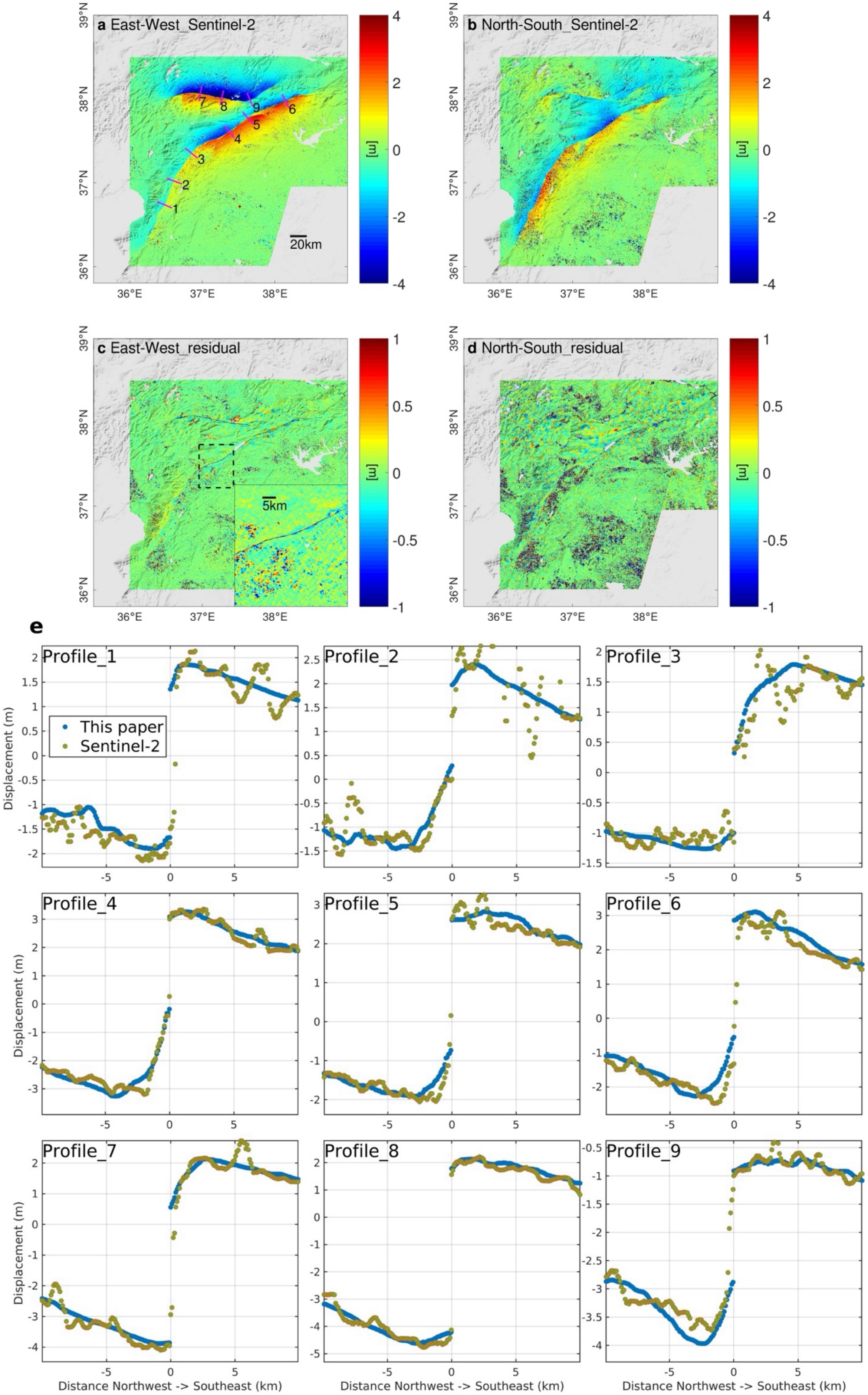

Supplementary Fig. 6 The comparison of displacements obtained by Sentinel-2 optical images in the Supplementary information of Ma et al. (2024)[4] and by SAR data in this paper. **a** and **b** are east-west (EW) and

north-south (NS) displacements obtained from Sentinel-2 images. **c** and **d** are the residuals of **a** and **d** with respect to the Sentinel-1 SAR-based EW and NS displacements derived here (i.e., Supplementary Figs. **1**a-b). The magenta lines with labeled number in **a** are selected profiles, and **e** shows the fault-parallel displacement comparison between the Sentinel-2 displacement and our SAR-based displacements. The insert panel in **c** is a zoom-in view of the dashed rectangle in **c**. It can be seen that our SAR-based horizontal displacements are well consistent with the Sentinel-2 optical results. Even if there is higher magnitude of residuals in the near-fault areas, the spatial extent is limited to only hundreds of meters, which has as negligible influence for our analysis of the up to 5 km width of the off-fault damage. The map background in **a-d** shows the elevation of the study region derived from the Shuttle Radar Topography Mission (SRTM) 3-arc seconds data[2].

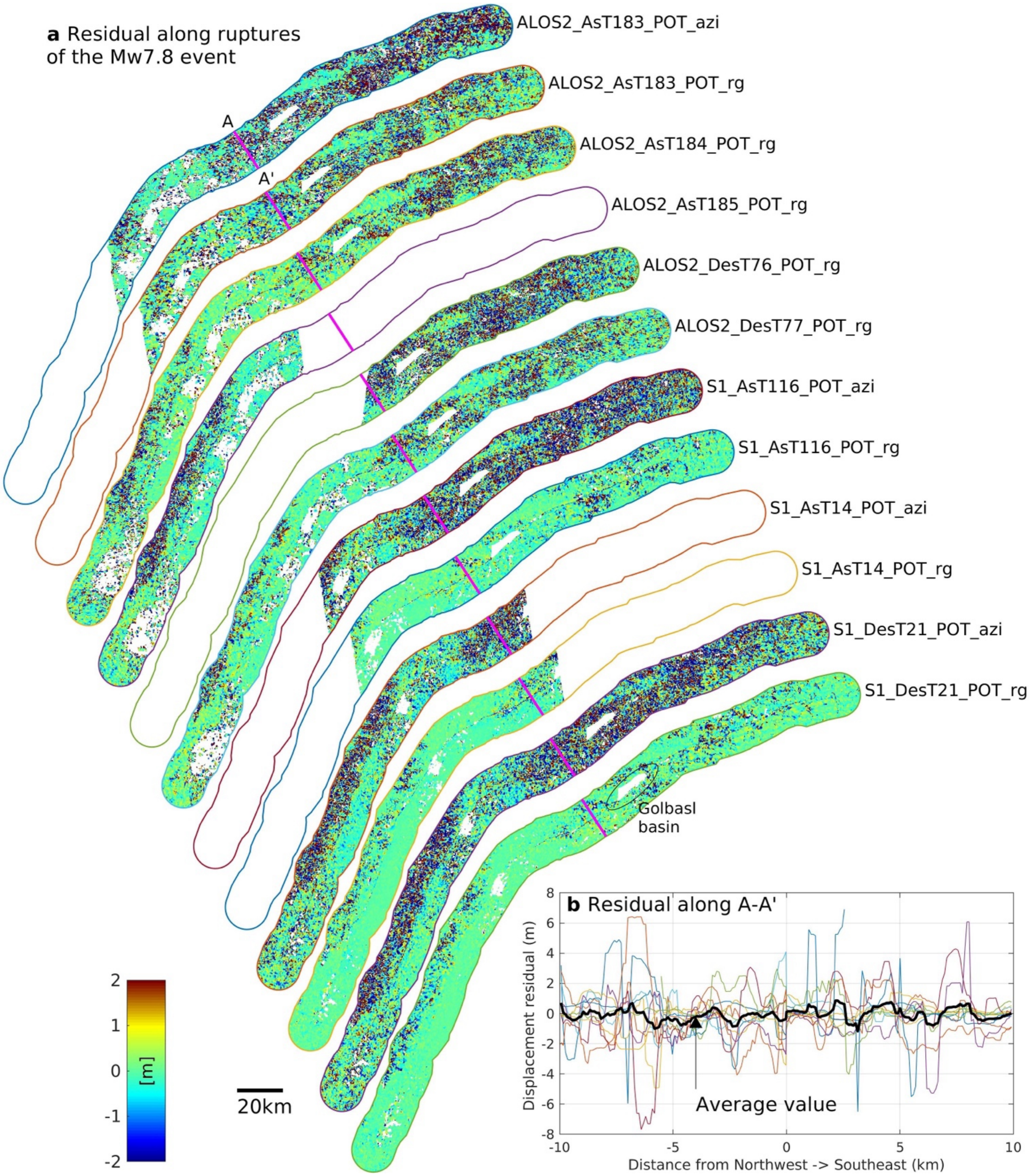

Supplementary Fig. 7. **a** Pixel-offset tracking (POT) residuals within a 10-km buffer zone along the ruptures of the Mw7.8 event. These residuals are obtained by differencing between the original observations and projected observations from the 3D displacements. **b** Residual values of the different observation sets along the profile A-A' in **a**, where the thin colored lines represent the corresponding observations in **a** with the same colored boundary, and the thick black line is the

average value of the colored lines. The location of x=0 in **b** represent the location of the fault. Although there are obvious residuals in these near-fault POT observations, there are no systematic deviations, suggesting that no single input data set is leading to a bias in the 3D displacement derivation. The residual maps of the standard differential InSAR (DInSAR), along-track multiple aperture interferometry (MAI), and range split-spectrum interferometry (RSSI) observations are not presented here since these observations are almost completely decorrelated in the near-fault regions. S1, Sentinel-1; As, ascending; Des, descending; T, track; azi, azimuth; rg, range.

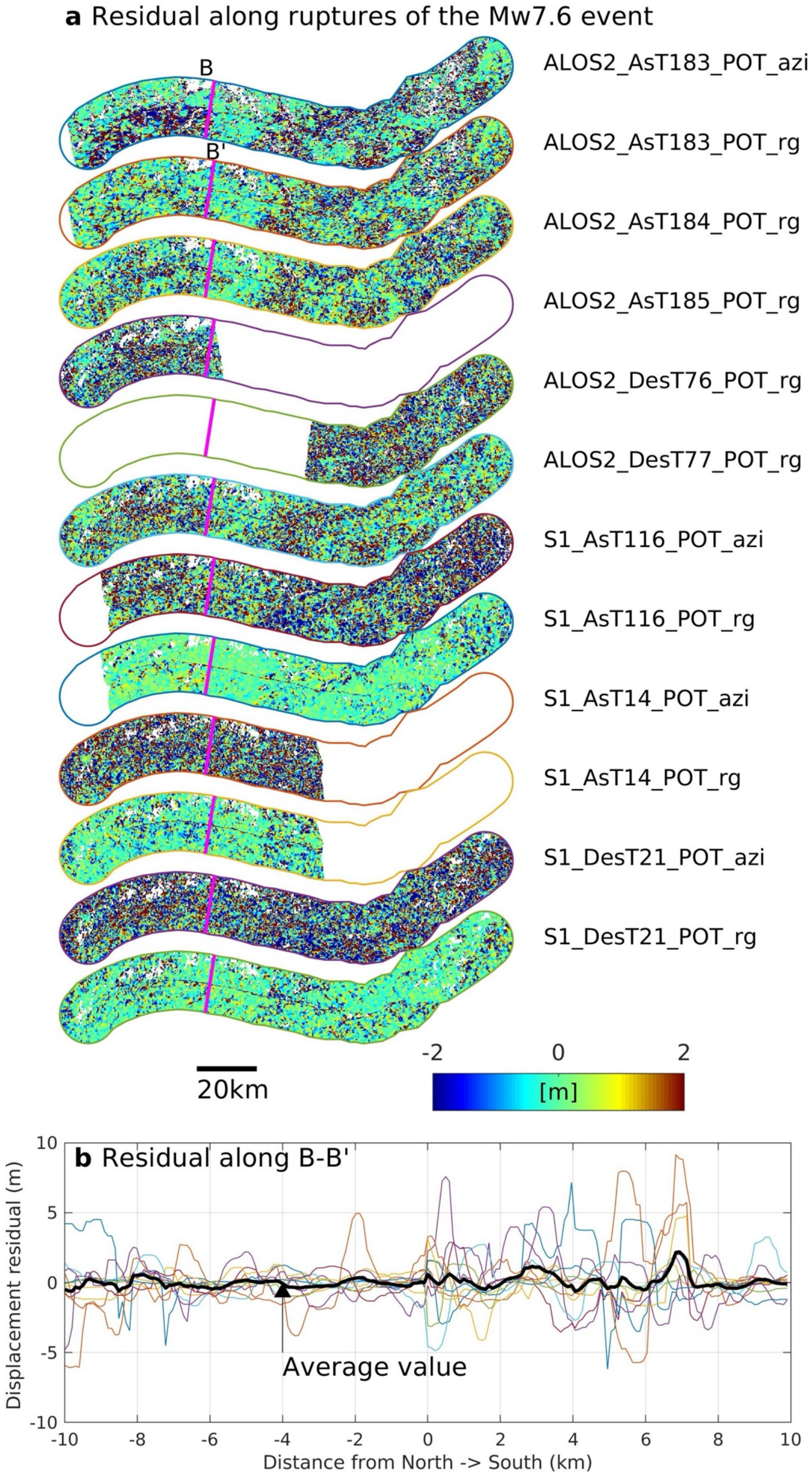

Supplementary Fig. 8. Same as Supplementary Fig. **7** but for the ruptures of the Mw7.6 event.

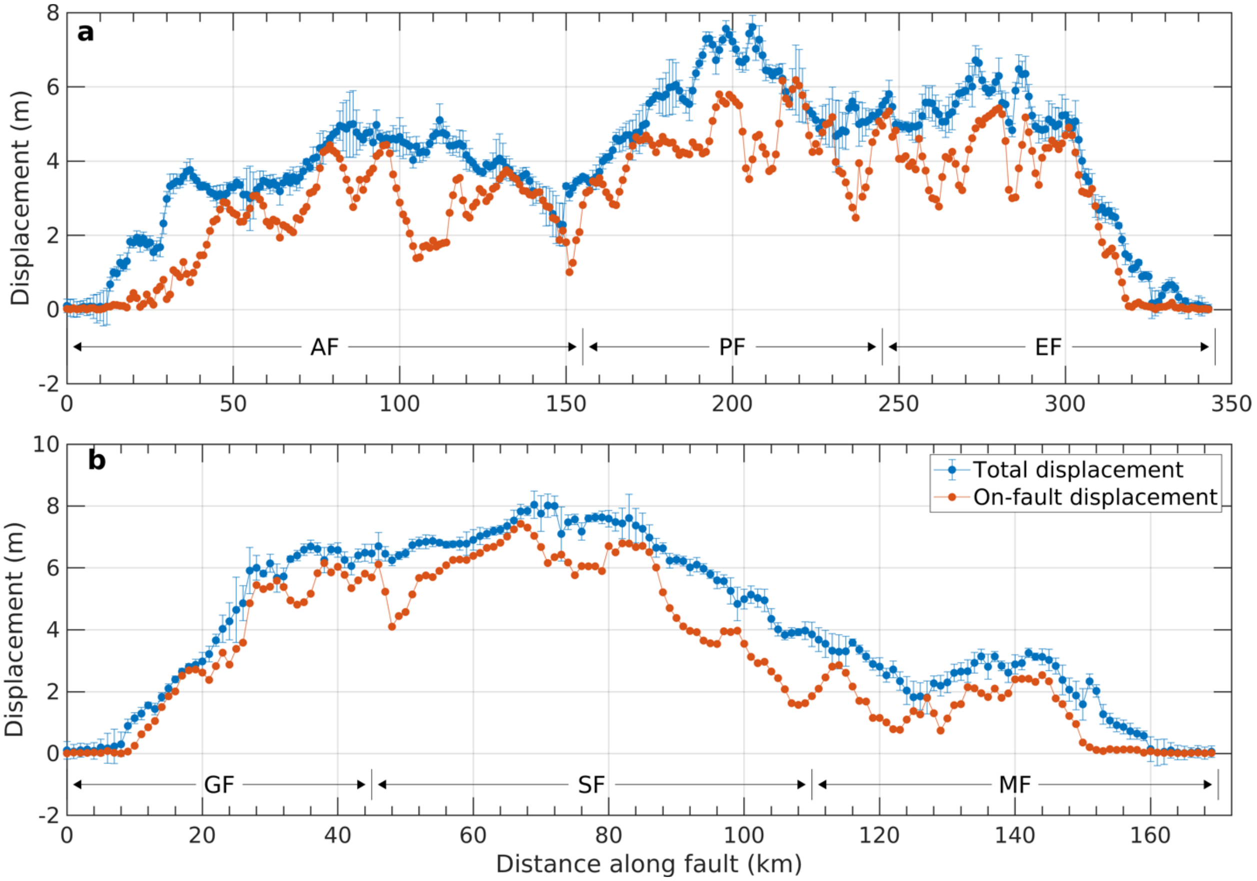

Supplementary Fig. 9 The estimated total displacement and the on-fault slip along the two main surface ruptures, the difference of which is the ASD amplitude. **a** The Mw7.8 event on the East Anatolian Fault. **b** The Mw7.6 event on the Sürgü fault. AF, Amanos Fault; PF, Pazarcık Fault; EF, Erkenek Fault; SF, Sürgü Fault; MF, Maraş Fault; GF, Göksun Fault. Slip peaks of the total displacement can be observed within segments of AF (5.0 m), PF (7.8 m), EF (6.0 m), SF (8.0 m), and MF (3.0 m).

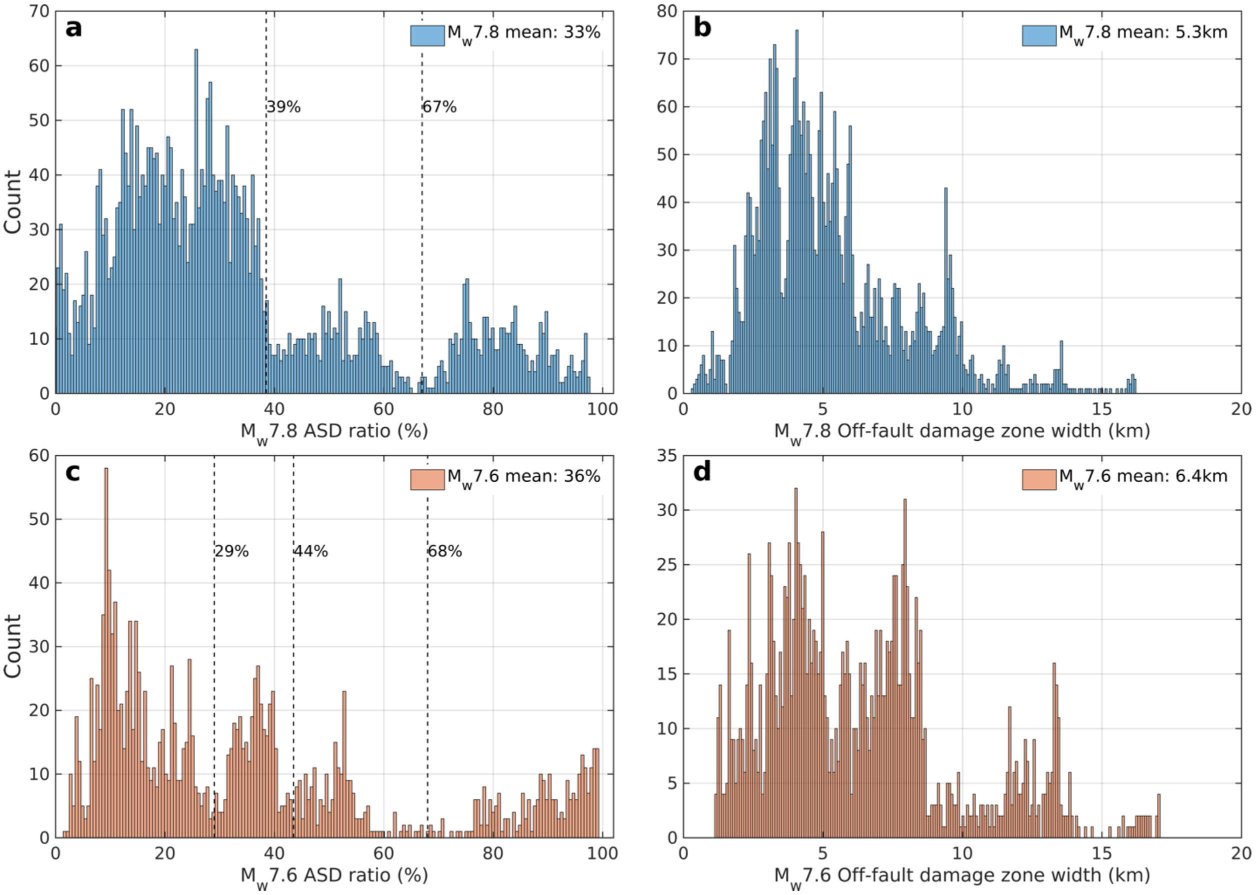

Supplementary Fig. 10 Histograms of the surface absent displacement (ASD) ratio (**a**, **c**) and the off-fault damage width (b, d) for the Mw 7.8 (**a**, **b**) and Mw 7.6 (**c**, **d**) events. The data in these histograms are same as the data in Fig. 3a in the main text. The number in the legend states the average value. Dashed vertical lines in **a** and **c** represent boundaries between local peaks. These boundary values were used for plotting the inset panel of Fig. 3a in the main text, which shows a distinct segment character of the ASD ratio that corresponds to variations in the geometrical complexity of the fault.

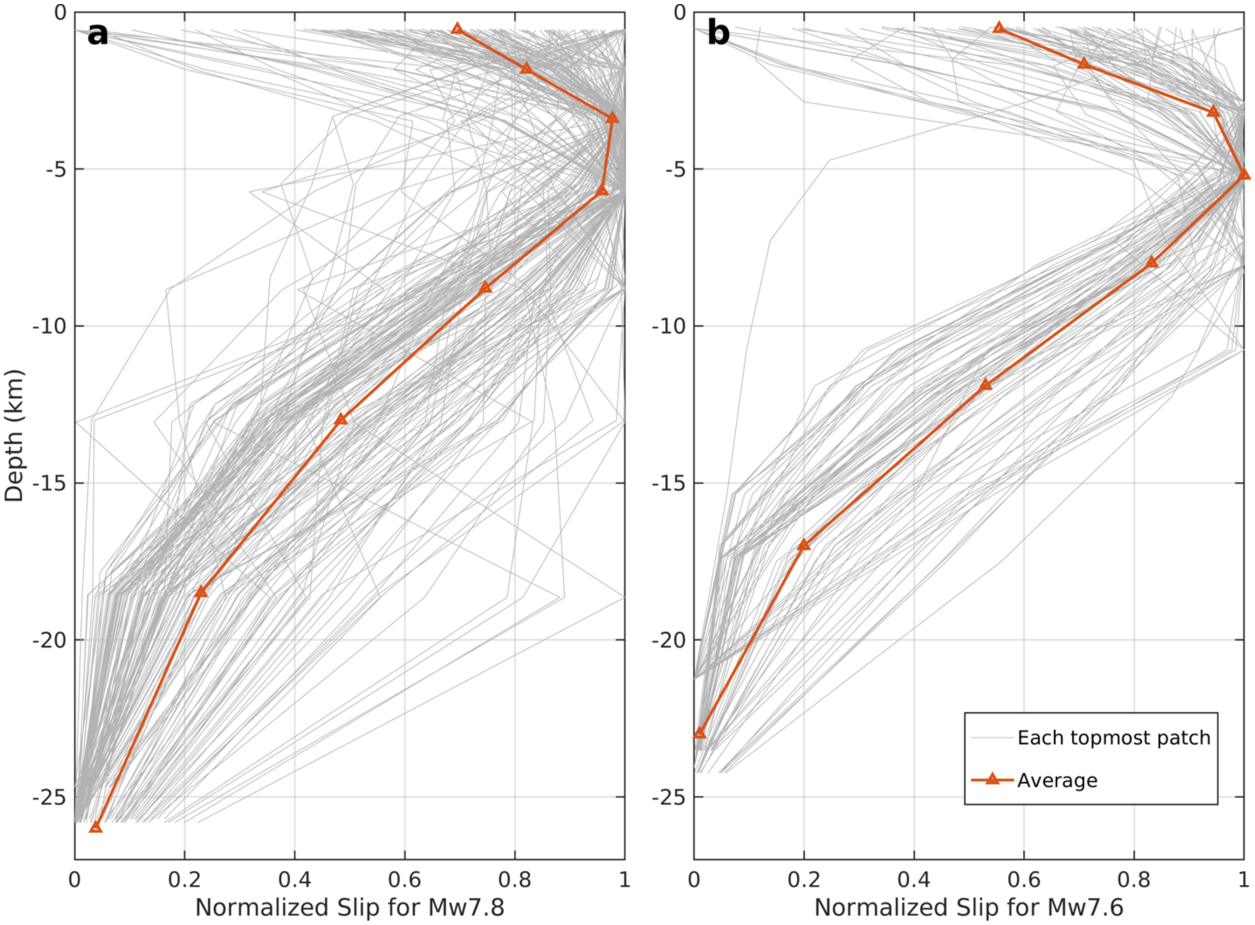

Supplementary Fig. 11 Normalized slip for different depths for each topmost patch of the slip model (Fig. 3b). **a** and **b** are for the Mw 7.8 and Mw 7.6 events, respectively. Each grey line was normalized by the maximum slip value at that location. The red line is the average of all the grey lines.

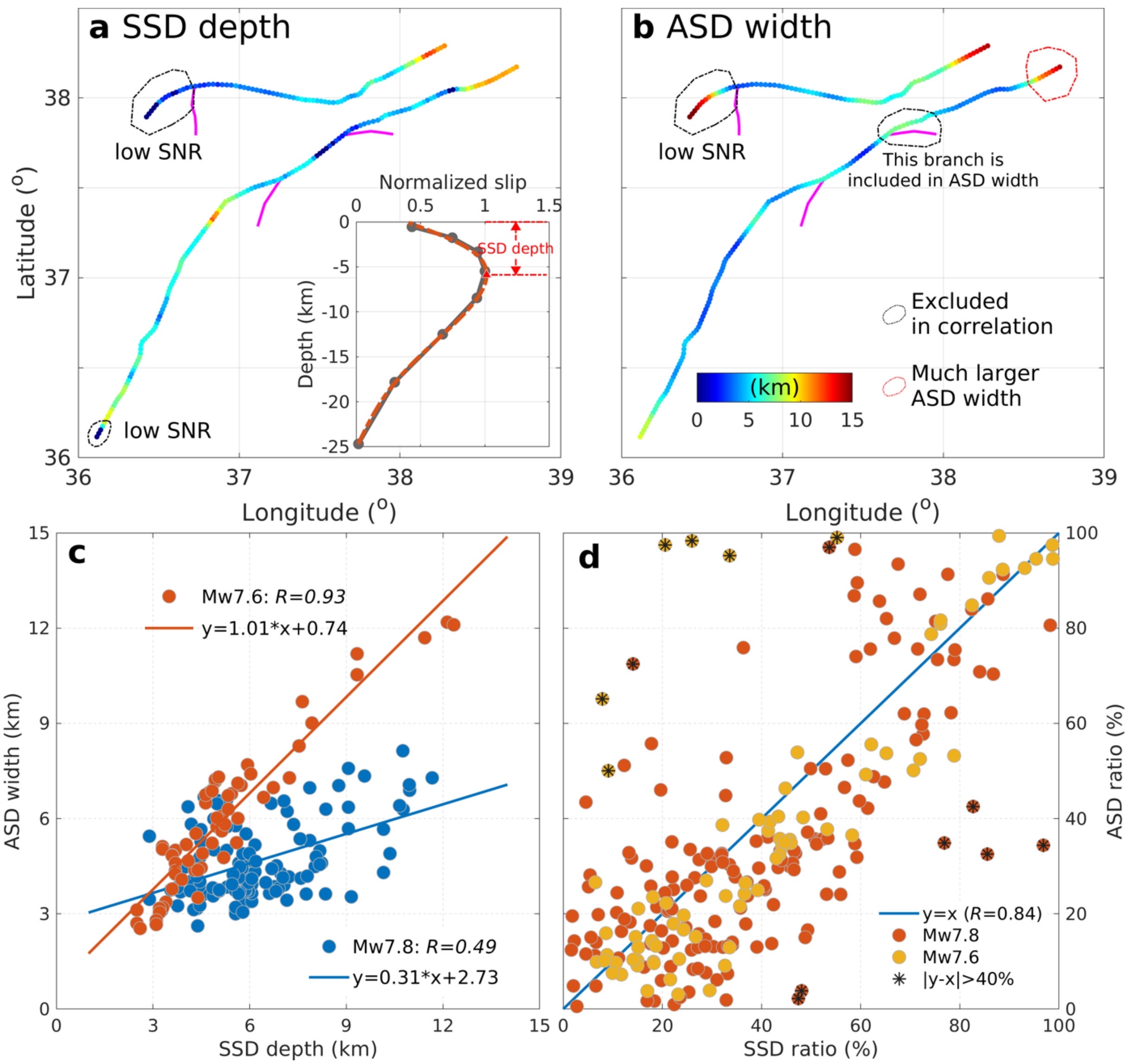

Supplementary Fig. 12 Correlation between shallow slip deficit (SSD) and absent surface displacement (ASD). **a** and **b** show the variations of SSD depths and ASD widths, respectively. The SSD depth is calculated as shown in the inset of **a**, where the grey dots are slip values on discrete patches inverted based on the elastic half-space model, and the red curve is a fitted spline. The depth corresponding to the maximum slip is the SSD depth. The overall spatial variations of SSD depths and ASD widths are consistent, except for the areas marked by black dashed polygons, where the signal-to-noise ratio (SNR) of the surface displacement is relatively low (i.e., at the western end of the second event and at the southwestern end of the first event) or where the ASD width includes a secondary fault branch modelled separately in the slip inversion. These areas (i.e., within the polygons) were excluded in the correlation analysis in **c**. Although the SSD depths and ASD widths in the red polygon of the first event are visually correlated, the latter is much larger than the former. **c** Scatter plot of ASD widths and SSD depths. The different slopes of the ASD widths versus SSD depths for two events may be attributed to different fault dip angles, i.e., that compared with a vertical fault, a dipping fault will result in a larger ASD width for the same SSD depth (**Supplementary Fig. 13**). **d** Scatter plot between ASD ratios and SSD ratios. Black asterisks

reflect the condition where |SSD ratio-ASD ratio|>40%, i.e., the magenta circles in Fig. 3b in the main text.

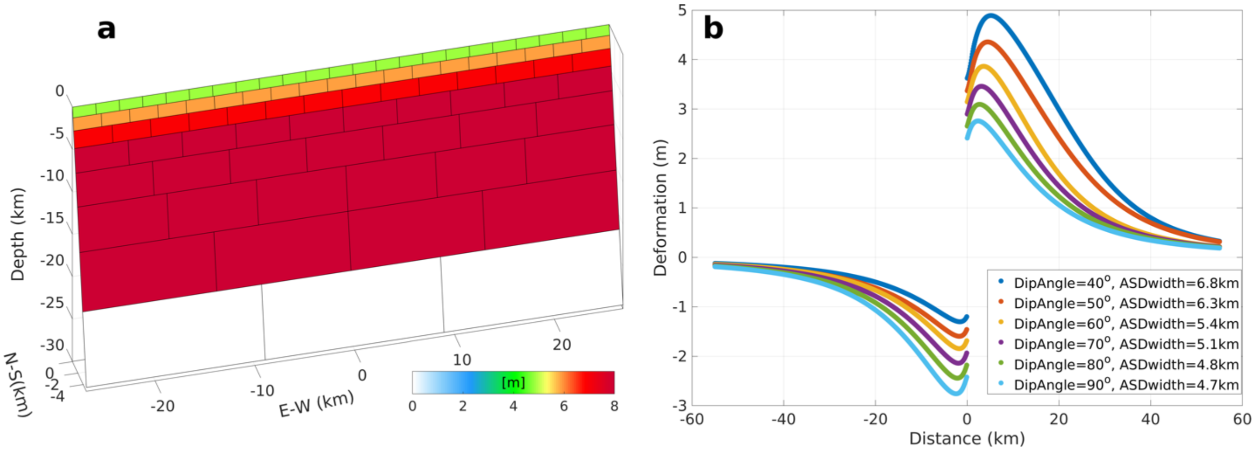

Supplementary Fig. 13 Fault-parallel surface displacements for different fault dip angles for faults with shallow slip deficit (SSD). We conducted several calculations to illuminate the change of the absent surface displacement (ASD) width with respect to the dip angle of the fault plane. On the fault, slip values in the 5-23 km depth range are 8 m, whereas from 5 km depth to the surface, the slip decreases to 4.8 m, corresponding to a SSD ratio of 40%. Then, we calculated several scenarios for fault dip angles changing from 90° to 40°, and panel **a** shows one of the cases with a dip angle of 80°. **b** The fault-parallel surface displacements for different dipping angles. Based on these displacement profiles, we estimated the ASD width of each case. As can be seen, under the same SSD depth and SSD ratio, the ASD width decreases with increasing dip angle. This is consistent with different slopes of the ASD width versus SSD depth for two mainshocks in Supplementary Fig. **12**c, with the Mw7.6 event having oblique slip on dipping faults yielding a steeper slope in the scatter plot of the ASD width versus SSD depth compared with the Mw7.8 event. Note that the ASD width in the legend of **b** includes both sides of the fault although the ASD width has different trends with the dip angle on two sides of the fault.

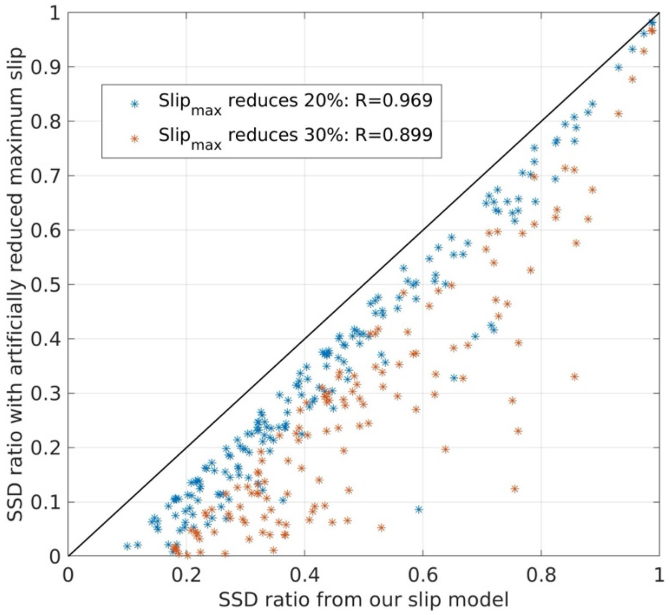

Supplementary Fig. 14 Comparison of the shallow slip deficit (SSD) ratio under the case that the maximum slip ($Slip_{max}$) value at depth is overestimated by 20% (blue) and 30% (red) due to using a simple elastic half-space model in the slip inversion[5]. In the legend, R represents the correlation coefficient. The black line is y=x. It can be seen that the SSD ratio values are highly correlated before and after correcting for a possible overestimation of the maximum slip, indicating that our simple homogenous elastic half-space model has little effect on the correlation analysis between the ASD and SSD ratios in this paper (i.e., Figs. 3c-d in the main manuscript, see Supplementary Text 1 for details).

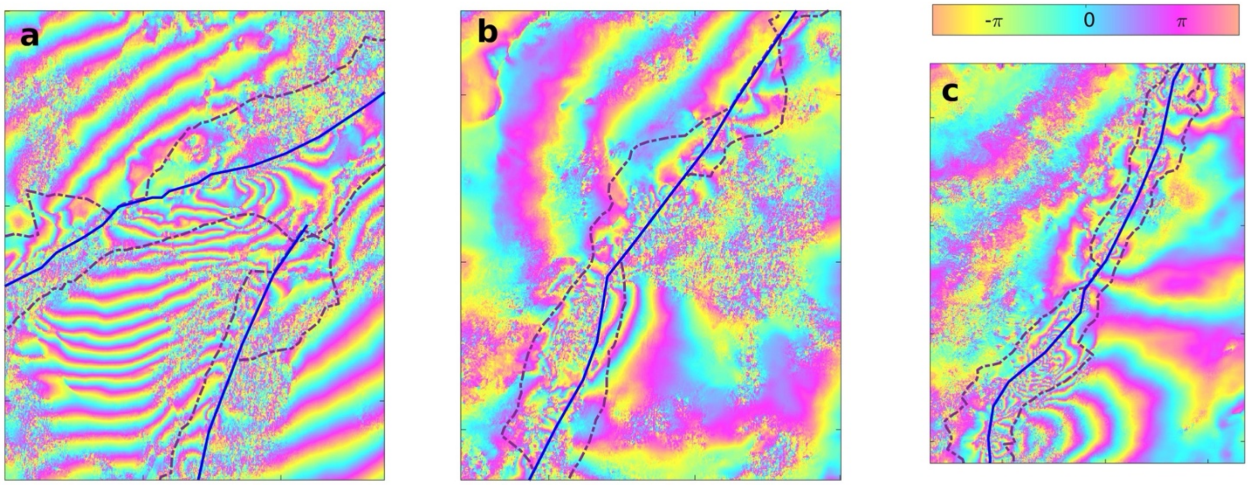

Supplementary Fig. 15 Same as Figs. 4d-f in the main text but without text, arrows, and fringe discontinuity annotations.

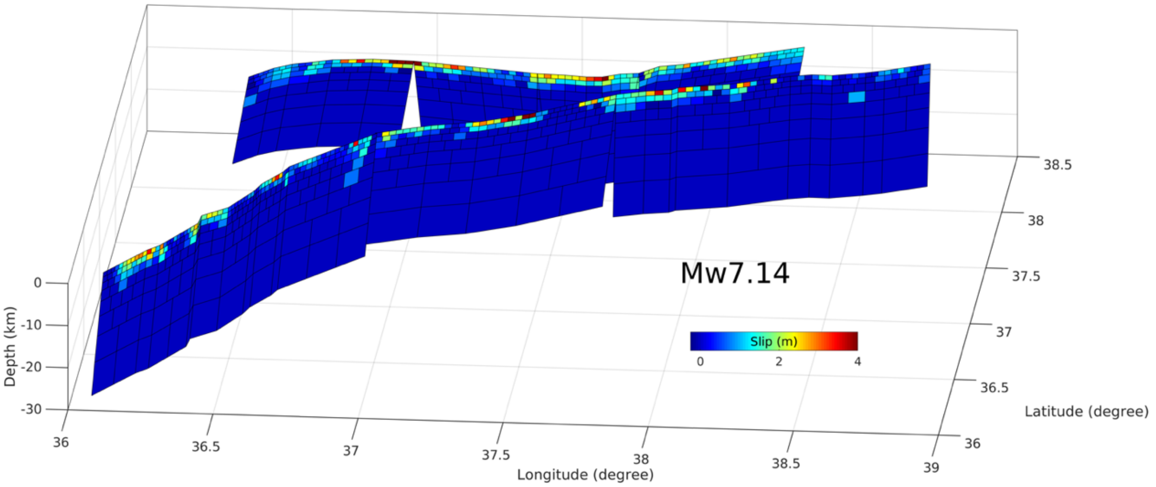

Supplementary Fig. 16 Estimated absent shallow slip on the two fault planes using the same fault geometry as for the coseismic slip model (Fig. 3b), with patches below (in blue) with no (zero) absent slip. The absent shallow slip values were obtained by differencing the local maximum of the modeled fault slip (at depth) and the slip on shallower fault patches. The estimated seismic moment of the absent shallow slip is 5.65×10^{19} Nm, which corresponds to a Mw 7.14 earthquake. This moment is far larger than that of recorded earthquakes, excluding the possibility that the earthquakes during the past century contributed significantly to the observed ASD.

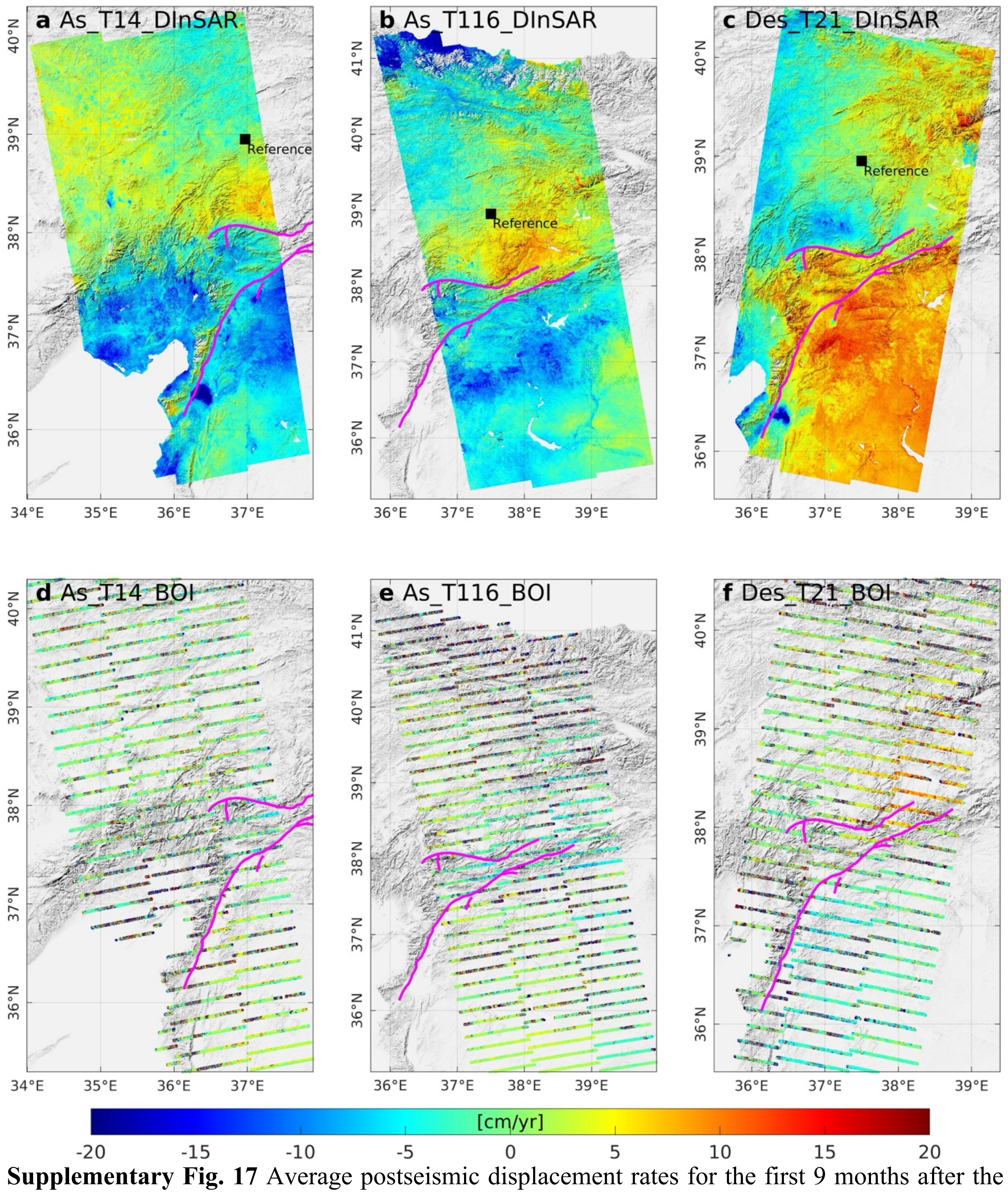

Supplementary Fig. 17 Average postseismic displacement rates for the first 9 months after the earthquakes, based postseismic Sentinel-1 SAR images. **a**, **b**, and **c** are the InSAR line-of-sight displacement rates for ascending tracks 14 and 116, and descending track 21, respectively. **d**, **e**, and **f** are the corresponding burst-overlap InSAR (BOI) azimuth displacement rates. The time span of the Sentinel-1 SAR images used here is 20230209-20231031, 20230228-20231107, and 20230210-20231101 for the three tracks, using a date format of yyyymmdd. Magenta lines mark the fault surface ruptures mapped from SAR-based observations, and the black squares in a-c

indicate the reference location for each track. It can be seen that there is negligible postseismic shallow slip around most segments of the main ruptures except for the northeastern end of ruptures. However, this postseismic shallow slip around the northeastern end is far smaller than needed to catch up with the observed meter-scale absent surface displacement (ASD), as demonstrated in Supplementary Fig. **18**. Since the spatial extent of the postseismic displacements is notably larger than that of the coseismic displacement, most of the postseismic displacements are likely due to deep viscoelastic processes. The map background shows the elevation of the study region derived from the Shuttle Radar Topography Mission (SRTM) 3-arc seconds data[2].

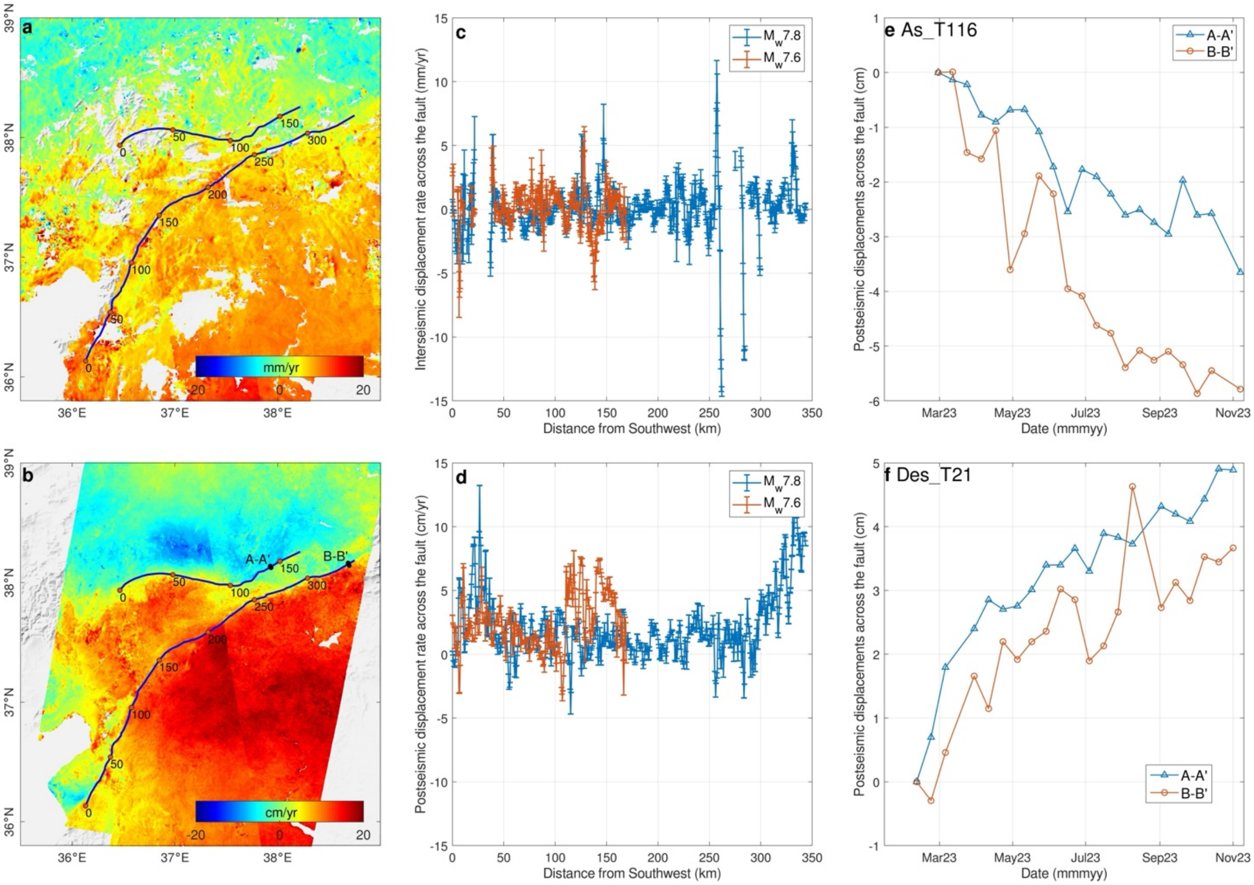

Supplementary Fig. 18 The displacement difference across the fault for the interseismic and postseismic periods. **a** is the interseismic east-west displacement rate from Weiss et al. (2020)[6] and **b** is the postseismic east-west displacement rate obtained in this study, where the blue lines represent the main coseismic ruptures mapped from SAR-based observations and the labeled numbers are the distance in km along the rupture from the southwest. In **b**, the black markers A-A' and B-B' at the northeastern ends of the ruptures are selected point pairs to show the difference of displacement time series across the rupture. Point pair A and A' (and B and B') are two points located on opposite sides and 1 km away from the rupture. **c** The difference of interseismic displacement rate across the fault along the two main ruptures, calculated by differencing the displacement rate value within 1.5 km range on each side of the rupture. **d** is same as **c** but for the

postseismic displacement rate. **e** The difference of postseismic displacement time series at point pairs A-A' and B-B' for the ascending T116 Sentinel-1 dataset. **f** is same as **e** but for the descending T21 Sentinel-1 dataset. In **c**, there is negligible difference of interseismic displacement rate between the two sides of the near-fault regions while **d** suggests 0-3 cm/yr shallow postseismic slip along most parts of the main ruptures, except at the northeastern end of the two ruptures, where it is 5-10 cm/yr. Although there is also an obvious contrast in displacement rate across the rupture at the southwestern end of the main rupture of Mw7.8 event, it mainly results from the 20th Feb. 2023 Mw6.3 aftershock. In **e** and **f**, we show the difference of postseismic displacement time series at the two selected point pairs A-A' and B-B', indicating a decaying shallow slip pattern around the eastern end of the main ruptures. This cm-level shallow slip can compensate only a small part of the missing meter-level coseimic absent surface displacement. The map background in **a-b** shows the elevation of the study region derived from the Shuttle Radar Topography Mission (SRTM) 3-arc seconds data[2].

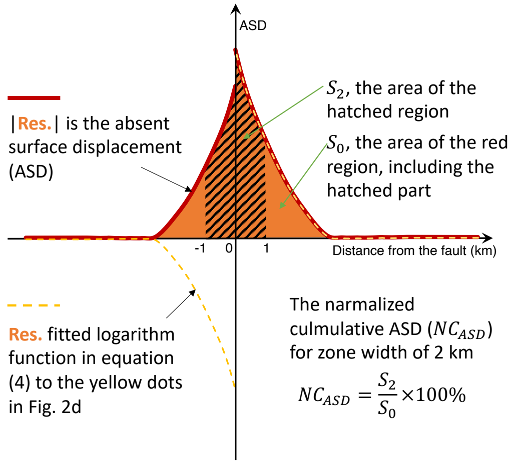

Supplementary Fig. 19 A schematic diagram illustrates the process of calculating the normalized cumulative ASD for a specific zone width of 2 km, as shown in Fig. 5. The dashed orange lines represent the logarithmic function fit (i.e., equation (4) in the main text) to the yellow dots in Fig. 2d. The red lines represent the absolute values of the dashed orange lines, corresponding to the ASD across the fault. The point at x=0 marks the location of the fault. For each fault-perpendicular profile, a single value of the normalized cumulative ASD (NC_{ASD}) can be calculated. The NC_{ASD} value for a 2 km zone width in Fig. 5 is obtained by averaging the NC_{ASD} values from all fault-perpendicular profiles along the main rupture of each event.

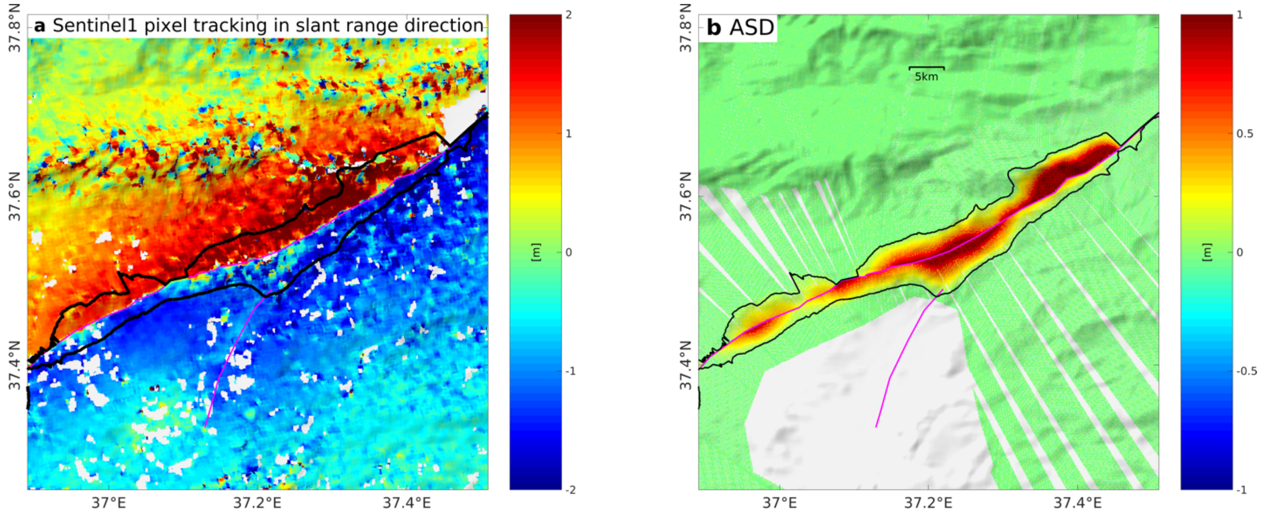

Supplementary Fig. 20 A zoom-in view of SAR pixel-offset tracking (POT) observations (S1_AsT116_POT_rg in **Supplementary Fig. 5**) to illuminate absent surface displacements (ASD) near the ruptures in the original SAR-based observations. **a** The original SAR POT observations. **b** The ASD estimated in this study for the same area. The large white region in **b** was masked during the estimation of the ASD along the main rupture to prevent influence from coseismic deformation on the splay fault. Magenta lines are the surface fault traces mapped from SAR-based observations and black lines outline the region of off-fault damage obtained in this paper. It can be observed that the displacement magnitude decreases near the rupture, and this decrease coincides with the estimated ASD. The displacement decrease does not result from the window-based POT method since the POT window size is only 500 m while the displacement decrease extends several km away from the fault. This shows that the ASD is directly seen in the original SAR POT observations. The map background shows the elevation of the study region derived from the Shuttle Radar Topography Mission (SRTM) 3-arc seconds data[2].

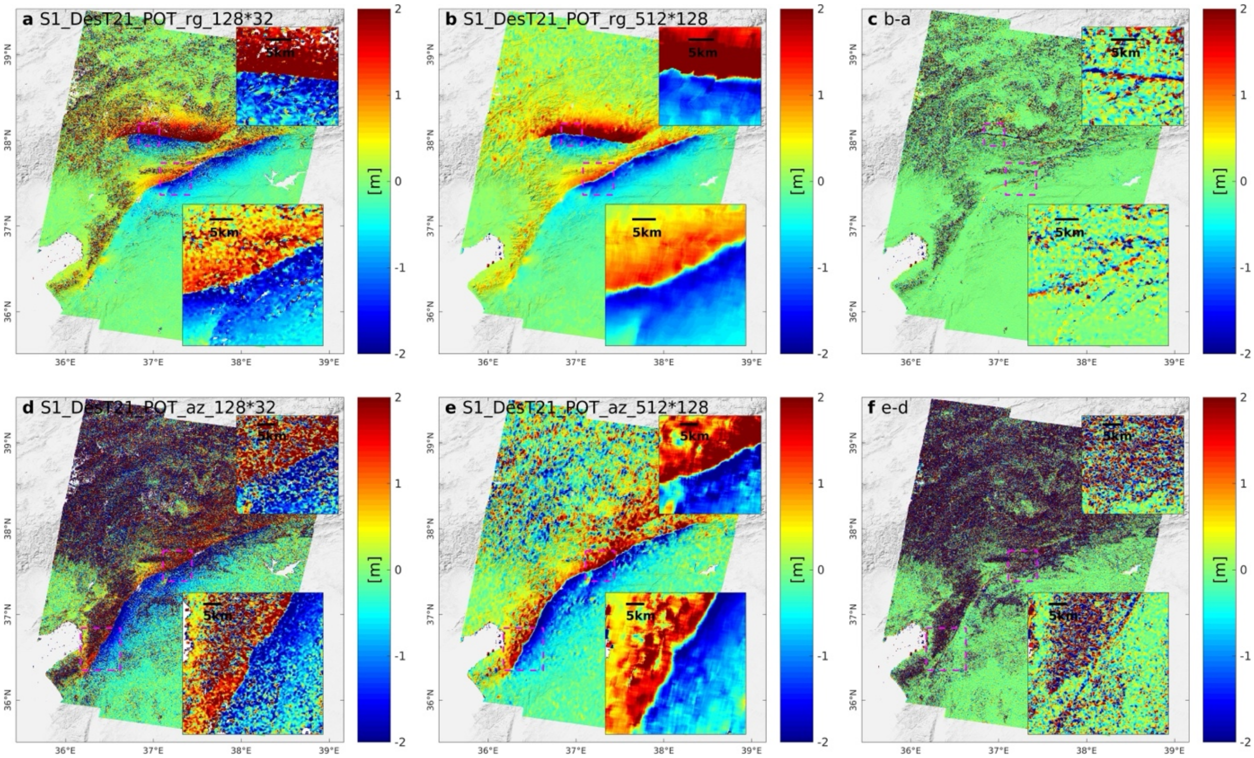

Supplementary Fig. 21 Pixel-offset tracking (POT) displacements estimated with different window sizes, i.e., 128*32 and 512*128 (range*azimuth), for descending (Des) Sentinel-1 (S1) track 21 (T21) images. azi, azimuth; rg, range. It can be seen that the main difference between different window sizes is the smoothness of the derived displacement, i.e., larger windows smoothen high-frequency noise. However, in the near-fault areas, the large window also smoothens the sharp displacement offset across the fault, which can bias our off-fault damage analysis. Therefore, we prefer to use the smaller correlation window for the pixel-offset tracking process to preserve the displacement details as much as possible. The map background shows the elevation of the study region derived from the Shuttle Radar Topography Mission (SRTM) 3-arc seconds data[2].

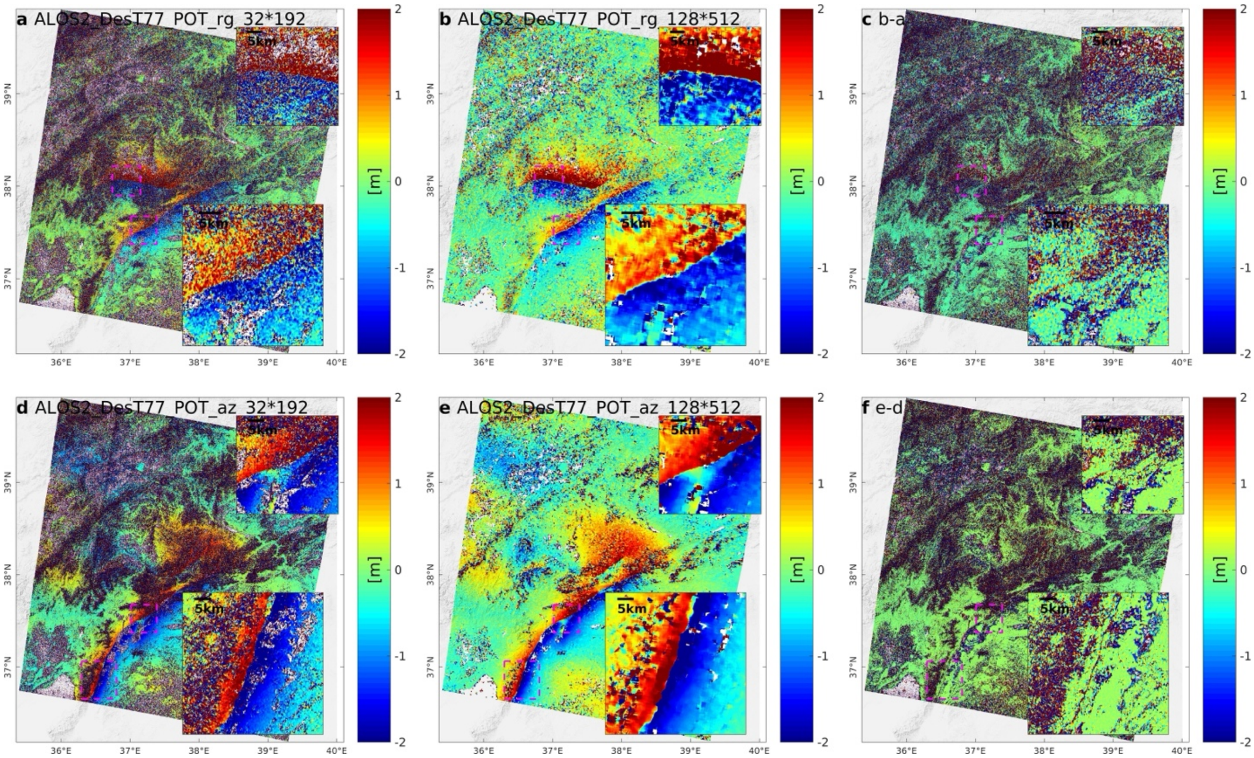

Supplementary Fig. 22 Same as Supplementary Fig. **21**, but for descending (Des) ALOS2 track 77 (T77) images. The map background shows the elevation of the study region derived from the Shuttle Radar Topography Mission (SRTM) 3-arc seconds data[2].

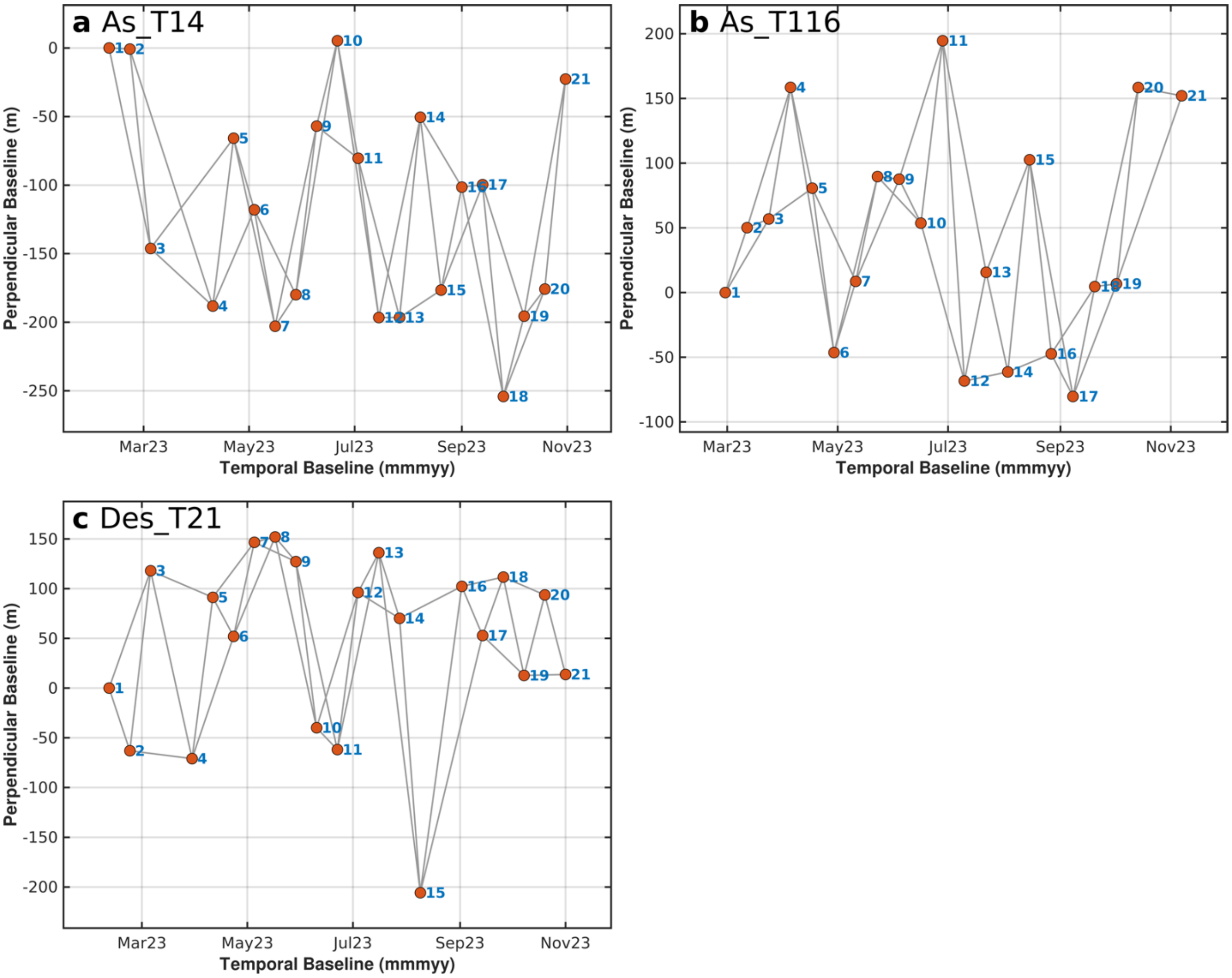

Supplementary Fig. 23 The spatio-temporal baselines of the postseismic Sentinel-1 SAR interferograms used in this study. **a**, **b**, and **c** are for the ascending track 14, ascending track 116, and descending track 21, respectively. Red circles represent the SAR images relative to the reference image (i.e., the first SAR image of each track obtained after the earthquakes), numbers by the circles indicate the SAR-image indices, and grey lines between circles the interferograms used in this study.

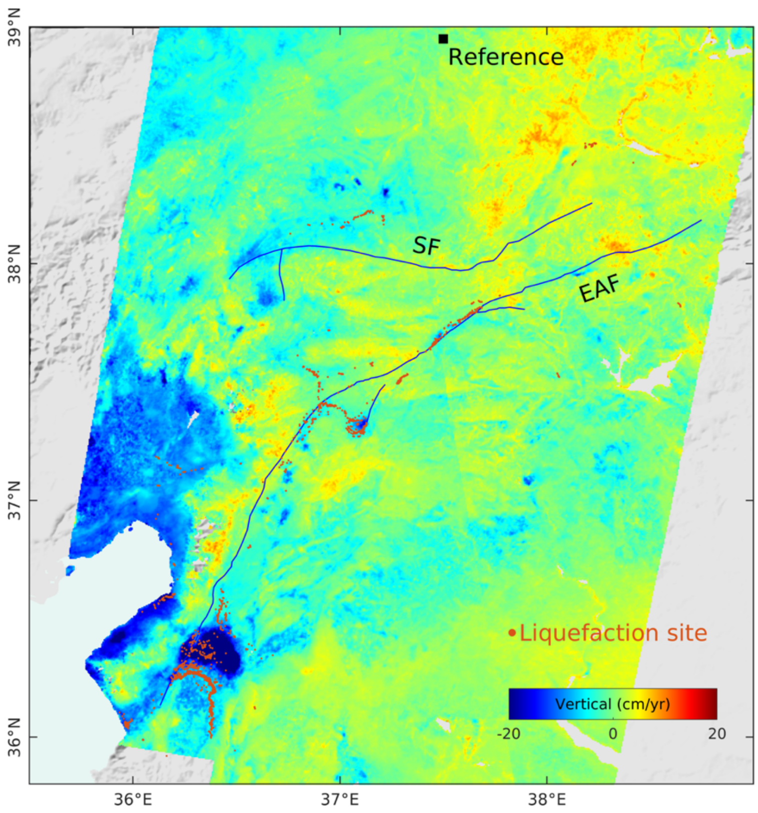

Supplementary Fig. 24 The average vertical postseismic displacement rate obtained from time-series interferometry of 9-month postseismic Sentinel-1 SAR images in this paper. The vertical displacements are not as strong as the east postseismic displacements (Fig. 4b in the main text). There are several local subsidence areas that are correlated with the postseismic liquefaction sites obtained in Taftsoglou et al. (2023)[7]. The map background shows the elevation of the study region derived from the Shuttle Radar Topography Mission (SRTM) 3-arc seconds data[2].

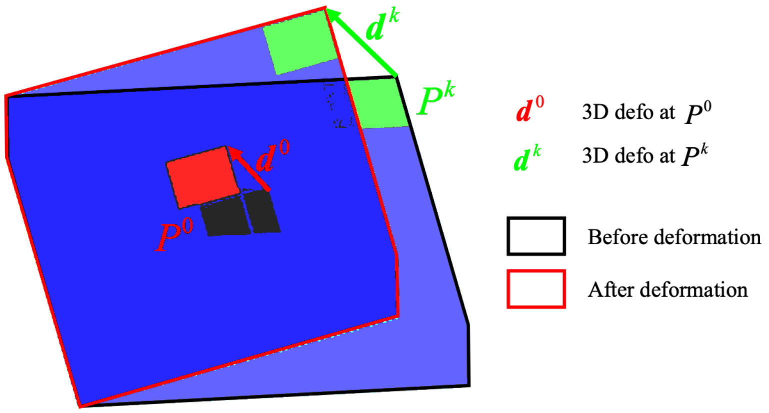

Supplementary Fig. 25 A schematic diagram of the strain model illustrates how the deformation of adjacent points is described to enhance understanding of the SM-VCE (Strain Model and Variance Component Estimation) method[8] used in this study to obtain coseismic three-dimensional (3D) surface displacements. Generally, deformation at different points within a window is correlated, and the strain model assumes a constant deformation gradient within this area. In this approach, SAR observations (blue pixels) around a target point (red pixel) are used to estimate the

3D displacement components of the target, providing a more robust result than the standard weighted least squares method[9,10], which only incorporates SAR observations at the target point itself.

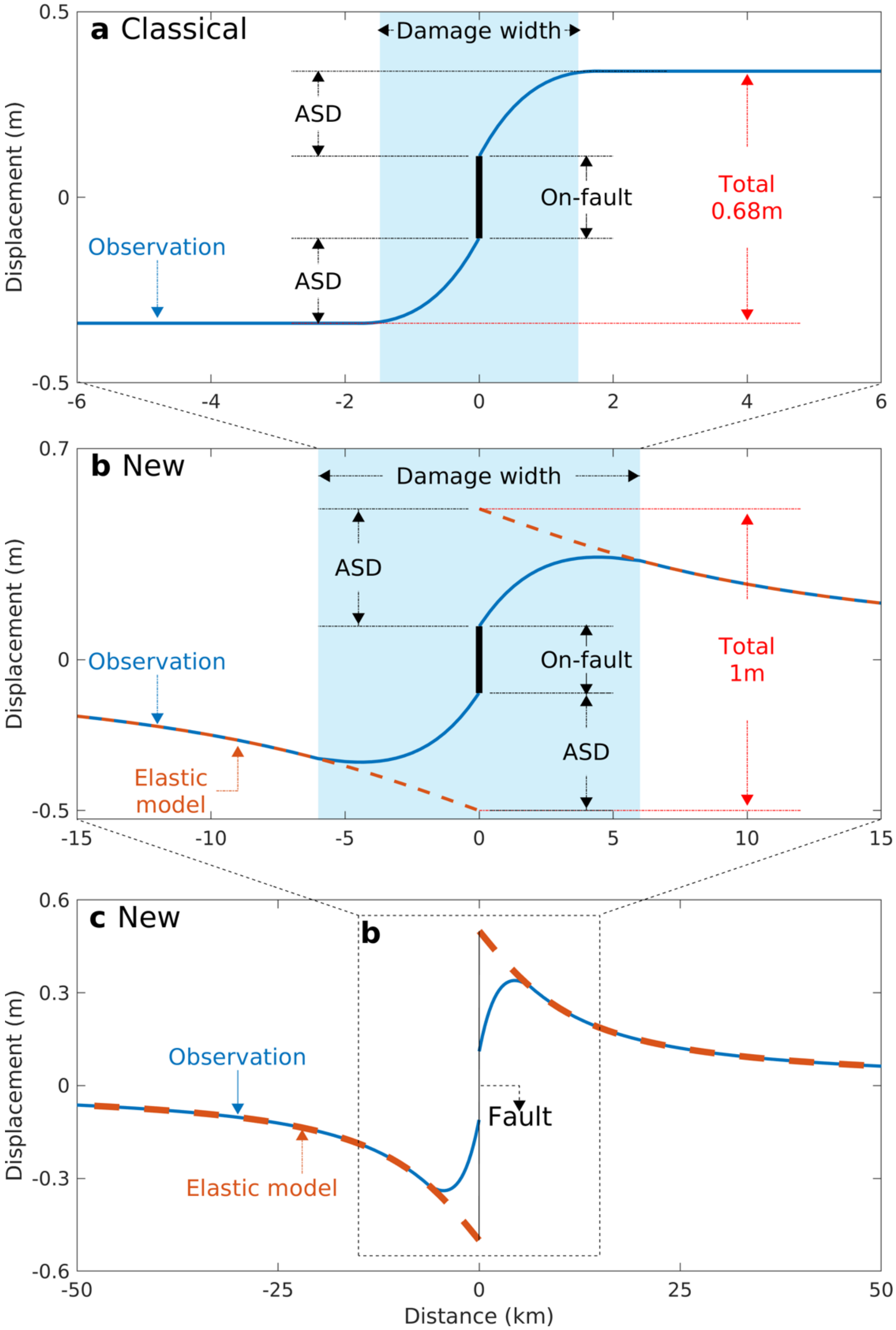

Supplementary Fig. 26 Comparison between the standard method in previous publications[11,12] and our new method for calculating the absent surface displacement (ASD). **a** The standard method,

in which the displacement values outside "the damage zone" are assumed to be constant and only observations from within several hundred meters or several km from the fault are considered (Supplementary Table 1). **b** and **c** demonstrate our new method, where almost the entire coseismic displacement field as well as elastic model predictions are involved for analysis. It can be seen that the standard method underestimates the magnitude of the total slip, the ASD, and the width of the off-fault damage.

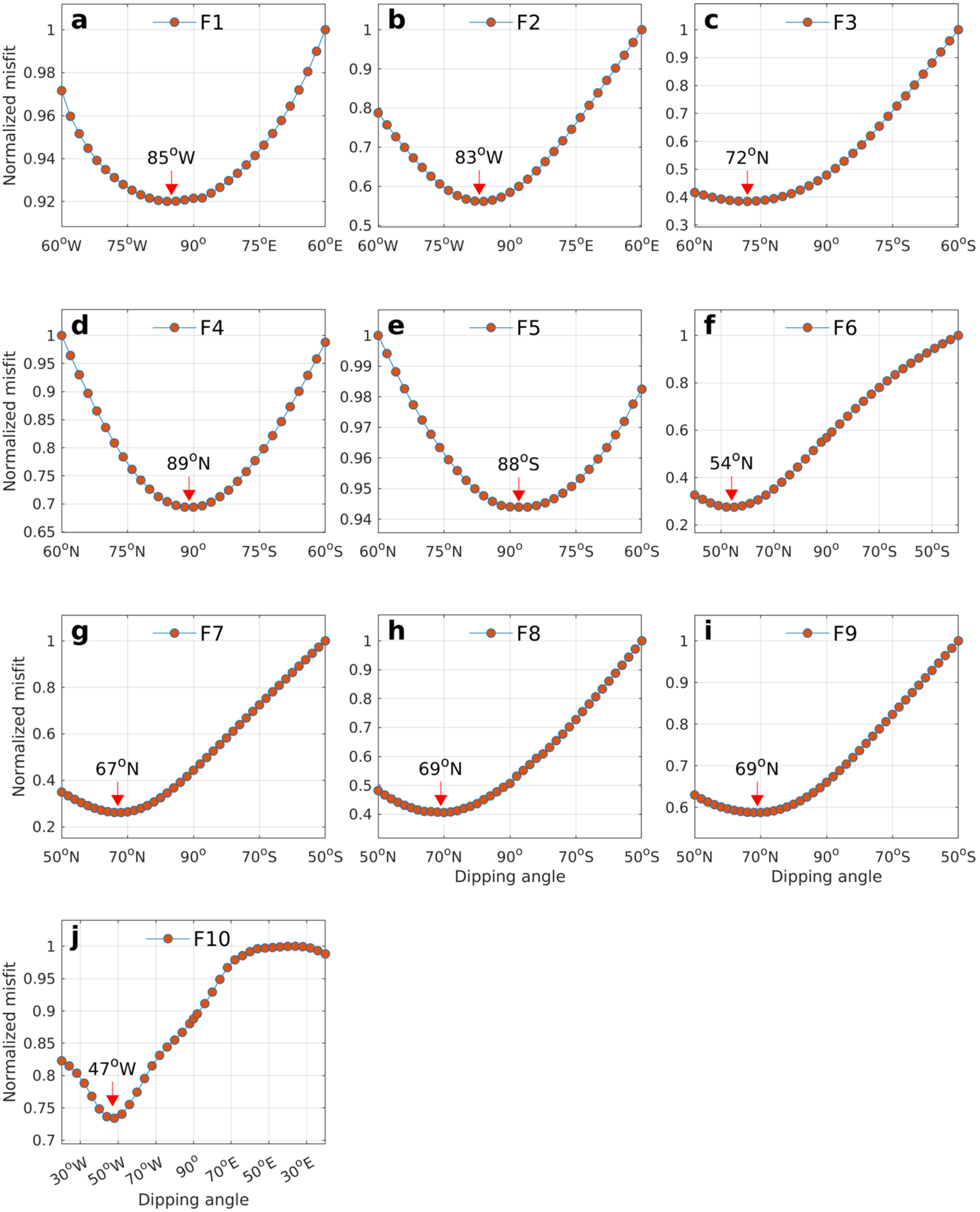

Supplementary Fig. 27 Normalized misfit versus different fault dip angles for fault segments F1-F10. The optimal dip angle was selected according to the minimum misfit for each fault segment. The suffix of the x-axis labels (i.e., E, W, N, or S) indicates the corresponding fault segment dip angle direction, i.e., towards the east, west, north, or south.

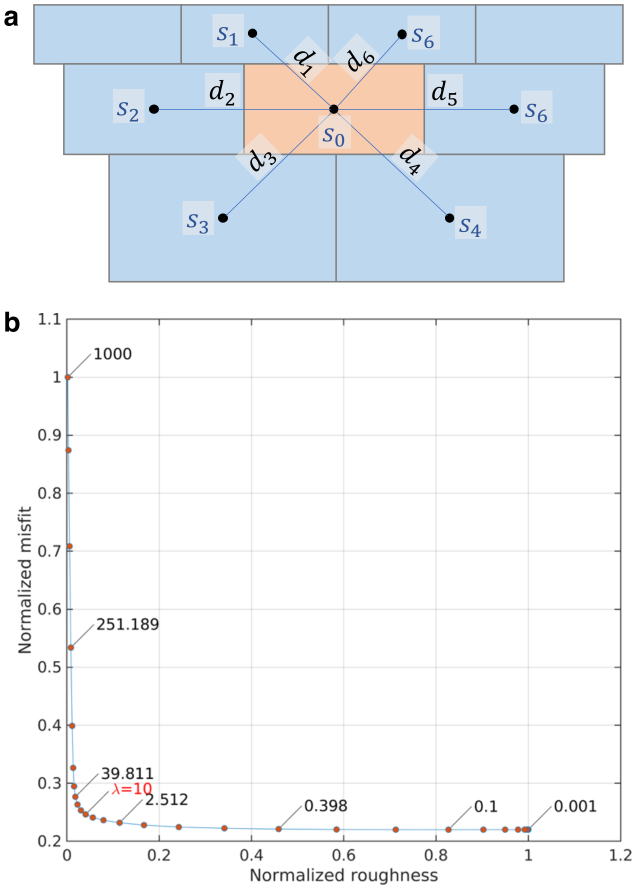

Supplementary Fig. 28 a A schematic diagram to illuminate the inverse distance-weighted smoothing constraint between adjacent patches during our slip model inversion. $d_i(i = 1,2,...,6)$ is the distance between the adjacent patch (blue) and the central patch (red). s_i and s_0 are the slip value of the blue and red patches, respectively. The smoothing constraint has the mathematical formula of $0 = s_0 - \sum_{i=1}^{6}(w_i s_i)$ ①, where $w_i = \frac{1}{d_i} \cdot \frac{1}{w_0}$ and $w_0 = \sum_{i=1}^{6} \frac{1}{d_i}$. If we have the Green function G between surface displacement observations L and the slip vector S for all patches, i.e., $L = G \cdot S$, we can estimate the slip vector as $\hat{S} = (G^{-1} \cdot G + \lambda \cdot H^{-1} \cdot H)^{-1} \cdot G^{-1} \cdot G \cdot L$, where H is the smoothing constraint matrix determined from ① and λ is the smoothing factor. **b** The trade-off curve between normalized fault-slip roughness and normalized misfit to the observations. The labeled numbers indicate the smoothing factor λ, where 10 was selected as the optimal one.

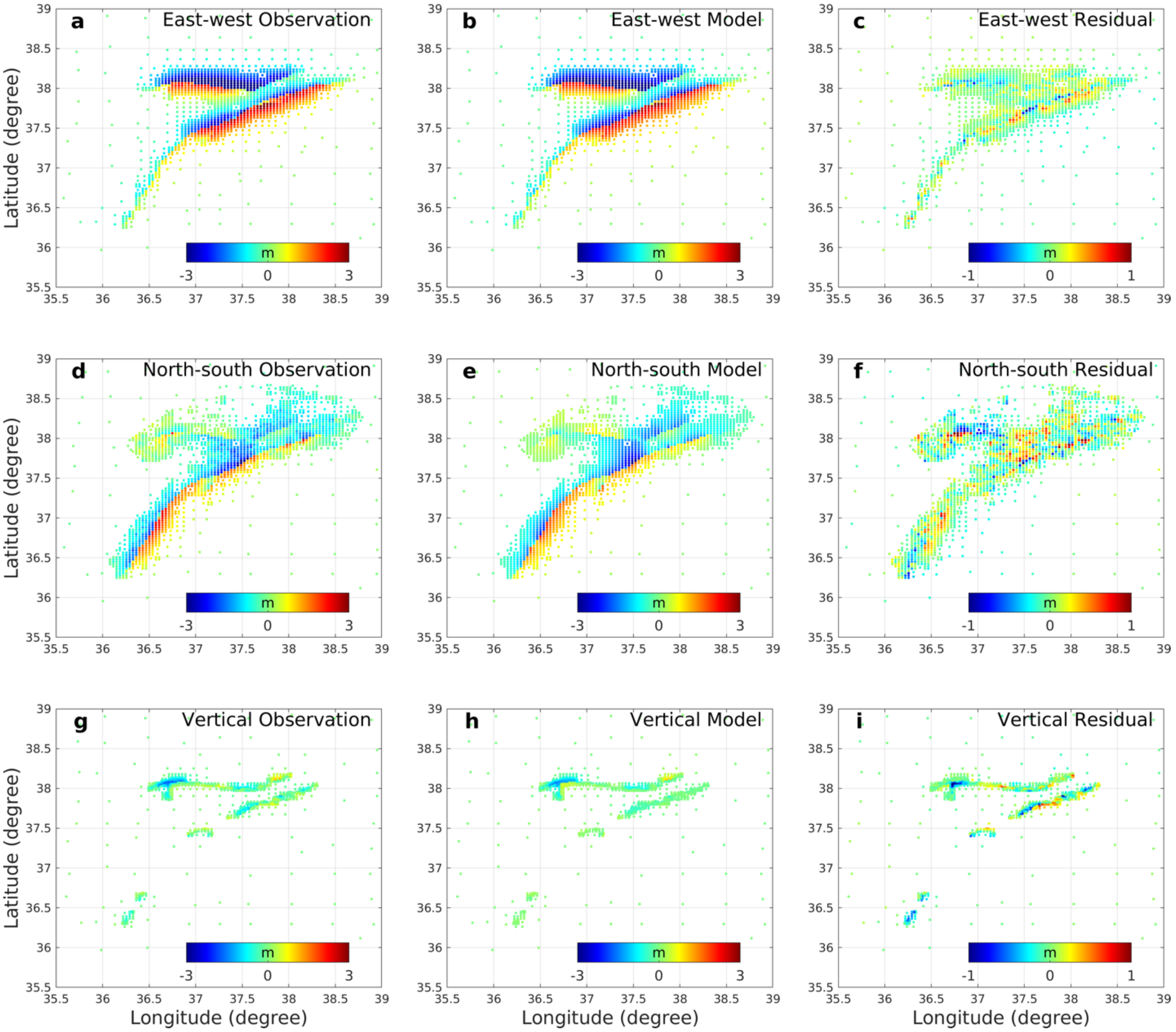

Supplementary Fig. 29 Quadtree-downsampled coseismic 3D surface displacements with **a-c**, **d-f**, and **g-i** being the east, north, and vertical components, respectively. **a, d,** and **g** are observations, **b, e,** and **h** are model predictions of the fault slip model in Fig. 3b, and **c, f**, and **i** are residuals between the observations and the model predictions.

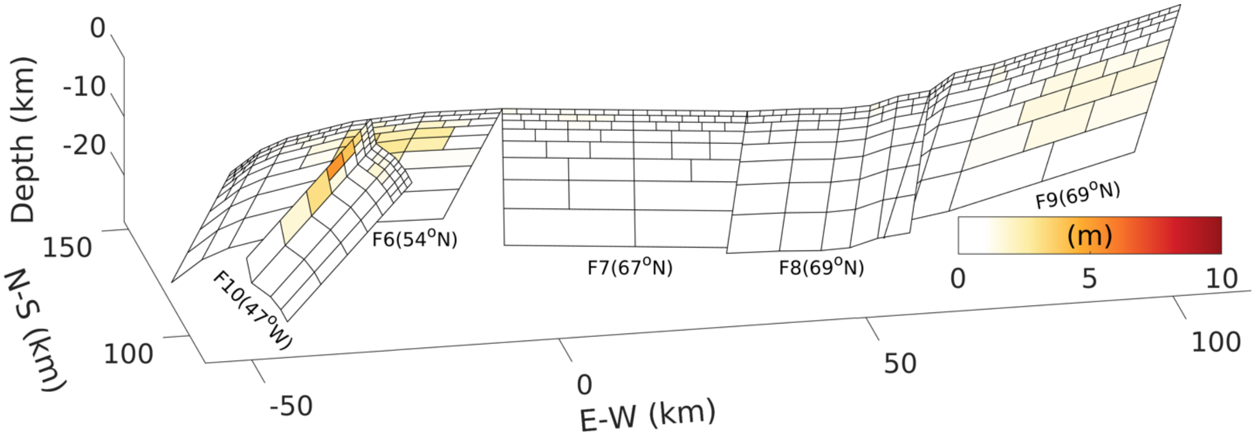

Supplementary Fig. 30 The estimated dip-slip component of the Mw 7.6 earthquake. The dip slip of the Mw 7.8 mainshock was considered negligible because of the limited vertical displacements near that fault. To compare this dip-slip solution with the strike-slip component (Fig. 3b), the colorbar limit here is set to [0, 10] m. It can be seen that the dip-slip values are much smaller than the strike-slip values, so we ignored the dip-slip component in the off-fault damage analysis. Note that the color representation here indicates the dip-slip magnitude, with segment F9 exhibiting reverse (thrust) faulting, while segments F6 to F8 and F10 normal faulting.

Supplementary Table 1 Information about previous earthquake case studies on off-fault damage based on remote sensing displacements.

Earthquake	Magnitude	Rupture length	Fault accumulative offset	Mean (maximum) coseismic strike-slip magnitude	Dataset (single image range)	Image spatial resolution	ASD ratio	Data range for analyzing damage	Off-fault damage width	Reference
The 1971 San Fernando California earthquake	Mw6.6	20 km	-	~2 m	Aerial stereo photograph (2km-3km)	1.1 m	69%	~2km	101m-131m	Gaudreau et al. (2023), JGR:SE[13]
The 1959 Hebgen Lake Earthquake	M7.2	36 km	-	~2 m	Aerial stereo-imagery (6km)	1.1 m	42.1%-59.7%	750m	47m-65m	Andreuttiova et al. (2022) GRL[14]
The 2013 Balochistan, Pakistan earthquake	Mw7.7	230km	6-11km	6.7m	Landsat-8 pixel tracking and WorldView-1/-2 (17.6 km x 14 km) for visual mapping	15m, 0.5m	45%	~2km	800m	Zinke et al. (2014) GGG[15]
The 2013 Balochistan, Pakistan earthquake	Mw7.7	~200km	6-11km	6-12 m	WorldView-1/-2 for visual mapping and pixel tracking (17.6 km x 14 km)	0.5m	28%	~8km	a few hundred meters of	Gold et al. (2015) Tectonophysics[16]

Earthquake	Magnitude	Rupture length	Fault accumulative offset	Mean (maximum) coseismic strike-slip magnitude	Dataset (single image range)	Image spatial resolution	ASD ratio	Data range for analyzing damage	Off-fault damage width	Reference
The 2013 Balochistan, Pakistan earthquake	Mw7.7	~200km	6-11km	6-12 m	Spot-5 (60km), WorldView2 (17.6 km x 14 km)	2.5m, 0.5m	-	3km	0-1.6km	Cheng et al. (2021) JGR:SE[17]
The 2013 Balochistan, Pakistan earthquake	Mw7.7	~200km	6-11km	6-12 m	WorldView (17.6 km x 14 km) and Pléiades (4-6km)	0.5m, 0.5m	30%	4-5km	0.2-1.5km	Antoine et al. (2022) JGR:SE[18]
The 2021 Kunlun earthquake	Mw7.8	400km	-	(8m)	SPOT-1/4 (60km)	10m	-	40km	127m-1km	Jara et al. (2021)[19]
The 1992 Landers earthquake	Mw7.3	80km	3.5km	~1.5m	NAPP aerial photographs (8km*8km)	1m	46%	400m	154m	Milliner et al. (2015) GGG[20]
The 1999 Hector earthquake	Mw7.1	45km	3-7km	2.8m	NAPP aerial photographs (8km*8km)	1m	39%	1-3km	121m	Milliner et al. (2016) GRL[12]
The 2016 Kumamoto, Japan, earthquake	Mw7.0	40km	0.8-1.4km	>2m	Lidar (1.6 km)	2.5-3.5 pionts/m^2	36-62%	2km	~250m	Scott et al. (2018) JGR:SE[21]
The 2016 Kaikōura earthquake	Mw7.8	180km	-	~11m	Spot-6 (60km)	1.8m	-	~8km	~2km	Klinger et al. (2018) GRL[22]

Earthquake	Magnitude	Rupture length	Fault accumulative offset	Mean (maximum) coseismic strike-slip magnitude	Dataset (single image range)	Image spatial resolution	ASD ratio	Data range for analyzing damage	Off-fault damage width	Reference
The 2016 Norcia Earthquake	Mw6.6	~20km	-	~7cm	Differential Terrestrial Laser Scanning (~0.2km^2)	10^2-10^4 pionts/m^2	50%	50m	8m	Wedmore et al. (2019) GRL[23]
The 2018 Hualien earthquake	Mw6.4	~8km	-	~1m	Pléiades	0.5m	60%	7-8km	200+m	Kuo et al. (2019) SRL[24]
The 2019 Ridgecrest earthquakes	Mw7.1, Mw6.4	45km	-	1.69m (4.78m)	Spot-6 (60km)	1.5m	34%	1-2km	69m	Milliner et al. (2021) JGR:SE[25]
The 2019 Ridgecrest earthquakes	Mw7.1, Mw6.4	45km	-	1.69m (4.78m)	WorldView (17.6 km x 14 km)	0.5m	31%	8km	2km	Antoine et al. (2021) BSSA[11]
The 2019 Ridgecrest earthquakes	Mw7.1, Mw6.4	45km	-	1.69m (4.78m)	WorldView (17.6 km x 14 km)	0.5m	59%<br>35%	2km	<2.5km (including entire zone of mapped rupture traces)	Gold et al. (2021) GGG[26]
The 2019 Ridgecrest earthquakes	Mw7.1, Mw6.4	45km	-	1.69m (4.78m)	WorldView (17.6 km x 14 km)	0.5m	-	1km	63m	Barnhart et al. (2020) NG[27]

Earthquake	Magnitude	Rupture length	Fault accumulative offset	Mean (maximum) coseismic strike-slip magnitude	Dataset (single image range)	Image spatial resolution	ASD ratio	Data range for analyzing damage	Off-fault damage width	Reference
The 2021 Maduo earthquake	Mw7.4	170km	4-5km	(7m)	Sentinel-2 (110km)	10m	88%	4km	835m	Li et al. (2023) SRL[28]
The 2023 Kahramanmaraş earthquakes	Mw7.8, Mw7.6	350km, 160km	33km	(8.5m)	Sentinel-2 (110km)	10m	54% 47%	20km	466m 700m	Provost et al. (2024) Scientific Report[29]

Supplementary Table 2 Information about the SAR images used to map the coseismic deformation in this study.

Sensor	Track	Date (yyyymmdd)	Perpendicular baseline (m)	Wavelength (cm)	Imaging mode
Sentinel-1	Ascending T14	20230128-20230209	171.1	5.6	TOPS
Sentinel-1	Ascending T116	20230204-20230228	-108.1	5.6	TOPS
Sentinel-1	Descending T21	20230129-20230210	-105.9	5.6	TOPS
ALOS2	Ascending T183	20190918-20230215	-8.4	24.3	ScanSAR
ALOS2	Ascending T184	20220905-20230220	10.6	24.3	ScanSAR
ALOS2	Ascending T185	20220910-20230211	259.9	24.3	ScanSAR
ALOS2	Descending T76	20220911-20230212	521.2	24.3	ScanSAR
ALOS2	Descending T77	20220916-20230217	-40.7	24.3	ScanSAR

Supplementary Discussion 1

Do geodetic slip inversions using simple homogeneous elastic half-space models significantly under- or overestimate shallow slip deficit?

Marchandon et al. (2021)[5] demonstrated that when inverting for fault slip with a simple homogenous elastic half-space model, it can artificially enhance the slip at depths of ~5 km. In this case, even if fault slip close to the surface is well-resolved by geodetic observations (i.e., SAR-based displacements in this paper), one may infer an apparent shallow slip deficit (SSD) from the slip model because the slip at depth is overestimated. Nevertheless, this overestimation of slip at depth has negligible effect on the correlation analysis between the absent surface displacement (ASD) and SSD ratios in Figs. 3c-d in the main paper. As demonstrated by the simulated experiments in Marchandon et al. (2021), the simplification of heterogenous underground elastic properties with a homogenous model would result in the overestimation of slip magnitude about 0.74 m between 3-6 km, which corresponds to about 20% of the input slip model at this depth. Here, we recalculated the SSD ratio for the 2023 Kahramanmaraş (Türkiye) earthquakes by artificially subtracting 20% from the maximum slip at depth of our slip model, aiming to account for the effect of the homogenous elastic model in overestimating the maximum slip at ~5 km depth. As shown in Supplementary Fig. 14, the SSD ratios are highly correlated (R=0.969) before and after subtracting an artificial slip value from the maximum slip value at depth. Even if we subtract

a larger proportion of the maximum slip (e.g., 30%), the correlation between two sets of SSD ratios is still very high. Besides of the SSD ratio, in the Fig. 3 of Marchandon et al. (2021), we can find that the SSD depth (i.e., the depth of maximum slip) seems unchanged with different underground elastic models. Therefore, we conclude here that using a homogenous elastic model has negligible effect on the estimation of the SSD ratio and on the correlation analysis between ASD and SSD ratios in this paper (i.e., Fig. 3c-d in the main text).

Reference

1. Liu J*, et al.* Three-dimensional surface displacements of the 8 January 2022 Mw6. 7 Menyuan earthquake, China from Sentinel-1 and ALOS-2 SAR observations. *Remote Sensing* **14**, 1404 (2022).

2. Farr TG*, et al.* The shuttle radar topography mission. *Rev. Geophys.* **45**, (2007).

3. Liu X, Zhao C, Zhang Q, Lu Z, Li Z. Deformation of the Baige landslide, Tibet, China, revealed through the integration of cross-platform ALOS/PALSAR-1 and ALOS/PALSAR-2 SAR observations. *Geophys. Res. Lett.* **47**, e2019GL086142 (2020).

4. Ma ZF*, et al.* Space Geodetic Insights to the Dramatic Stress Rotation Induced by the February 2023 Turkey-Syria Earthquake Doublet. *Geophys. Res. Lett.* **51**, e2023GL107788 (2024).

5. Marchandon M, Hollingsworth J, Radiguet M. Origin of the shallow slip deficit on a strike slip fault: Influence of elastic structure, topography, data coverage, and noise. *Earth Planet. Sci. Lett.* **554**, 116696 (2021).

6. Weiss JR*, et al.* High-resolution surface velocities and strain for Anatolia from Sentinel-1 InSAR and GNSS data. *Geophys. Res. Lett.* **47**, (2020).

7. Taftsoglou M, Valkaniotis S, Papathanassiou G, Karantanellis E. Satellite Imagery for Rapid Detection of Liquefaction Surface Manifestations: The Case Study of Tu(sic)rkiye-Syria 2023 Earthquakes. *Remote Sensing* **15**, 4190 (2023).

8. Liu J, Hu J, Li Z, Zhu JJ, Sun Q, Gan J. A method for measuring 3-D surface deformations with InSAR based on strain model and variance component estimation. *IEEE Trans. Geosci. Remote Sens.* **56**, 239-250 (2018).

9. Wright TJ, Parsons BE, Lu Z. Toward mapping surface deformation in three dimensions using InSAR. *Geophys. Res. Lett.* **31**, 169-178 (2004).

10. Jung HS, Lu Z, Won JS, Poland MP, Miklius A. Mapping three-dimensional surface deformation by combining multiple-aperture interferometry and conventional interferometry: Application to the June 2007 eruption of Kilauea volcano, Hawaii. *IEEE Geosci. Remote Sens. Lett.* **8**, 34-38 (2011).

11. Antoine SL, Klinger Y, Delorme A, Wang K, Burgmann R, Gold RD. Diffuse deformation and surface faulting distribution from submetric image correlation along the 2019 Ridgecrest, California, ruptures. *Bull. Seismol. Soc. Am.* **111**, 2275-2302 (2021).

12. Milliner CWD, Dolan JF, Hollingsworth J, Leprince S, Ayoub F. Comparison of coseismic near-field and off-fault surface deformation patterns of the 1992 Mw 7.3

Landers and 1999 Mw 7.1 Hector Mine earthquakes: Implications for controls on the distribution of surface strain. *Geophys. Res. Lett.* **43**, 10115-10124 (2016).

13. Gaudreau E, Hollingsworth J, Nissen E, Funning GJ. Complex 3-D surface deformation in the 1971 San Fernando, California earthquake reveals static and dynamic controls on off-fault deformation. *J. Geophys. Res. Solid Earth* **128**, e2022JB024985 (2023).

14. Andreuttiova L, Hollingsworth J, Vermeesch P, Mitchell TM, Bergman E. Revisiting the 1959 Hebgen Lake Earthquake Using Optical Image Correlation; New Constraints on Near-Field 3D Ground Displacement. *Geophys. Res. Lett.* **49**, e2022GL098666 (2022).

15. Zinke R, Hollingsworth J, Dolan JF. Surface slip and off- fault deformation patterns in the 2013 MW 7.7 Balochistan, Pakistan earthquake: Implications for controls on the distribution of near- surface coseismic slip. *Geochemistry Geophysics Geosystems* **15**, 5034-5050 (2014).

16. Gold RD, Reitman NG, Briggs RW, Barnhart WD, Hayes GP, Wilson E. On- and off-fault deformation associated with the September 2013 Mw 7.7 Balochistan earthquake: Implications for geologic slip rate measurements. *Tectonophysics* **660**, 65-78 (2015).

17. Cheng G, Barnhart WD. Permanent co-seismic deformation of the 2013 Mw7.7 Baluchistan, Pakistan earthquake from high-resolution surface strain analysis. *J. Geophys. Res. Solid Earth* **126**, e2020JB020622 (2021).

18. Antoine SL, Klinger Y, Delorme A, Gold RD. Off-Fault Deformation in Regions of Complex Fault Geometries: The 2013 Mw7.7, Baluchistan Rupture (Pakistan). *Journal of Geophysical Research-Solid Earth* **127**, e2022JB024480 (2022).

19. Jara J*, et al.* Signature of transition to supershear rupture speed in the coseismic off-fault damage zone. *P Roy Soc a-Math Phy* **477**, 1-23 (2021).

20. Milliner CWD, Dolan JF, Hollingsworth J, Leprince S, Ayoub F, Sammis CG. Quantifying near-field and off-fault deformation patterns of the 1992 M_w 7.3 Landers earthquake. (2015).

21. Scott CP, Arrowsmith R, Nissen E, Lajoie L, Maruyama T, Chiba T. The M7 2016 Kumamoto, Japan, Earthquake: 3-D Deformation Along the Fault and Within the Damage Zone Constrained From Differential Lidar Topography. *J. Geophys. Res. Solid Earth* **123**, 6138-6155 (2018).

22. Klinger Y*, et al.* Earthquake damage patterns resolve complex rupture processes. *Geophys. Res. Lett.* **45**, 10279-10287 (2018).

23. Wedmore LNJ, Gregory LC, McCaffrey KJW, Goodall H, Walters RJ. Partitioned off-fault deformation in the 2016 Norcia earthquake captured by differential terrestrial laser scanning. *Geophys. Res. Lett.* **46**, 3199-3205 (2019).

24. Kuo CH, Huang JY, Lin CM, Hsu TY, Chao SH, Wen KL. Strong ground motion and pulse-like velocity observations in the near-fault region of the 2018 Mw 6.4 Hualien, Taiwan, earthquake. *Seismol. Res. Lett.* **90**, 40-50 (2019).

25. Milliner C*, et al.* Bookshelf kinematics and the effect of dilatation on fault zone inelastic deformation: Examples from optical image correlation measurements of the 2019 Ridgecrest earthquake sequence. *J. Geophys. Res. Solid Earth* **126**, e2020JB020551 (2021).

26. Gold RD, DuRoss CB, Barnhart WD. Coseismic Surface Displacement in the 2019 Ridgecrest Earthquakes: Comparison of Field Measurements and Optical Image Correlation Results. *Geochemistry Geophysics Geosystems* **22**, e2020GC009326 (2021).

27. Barnhart WD, Gold RD, Hollingsworth J. Localized fault-zone dilatancy and surface inelasticity of the 2019 Ridgecrest earthquakes. *Nat. Geosci.* **13**, 699–704 (2020).

28. Li CL, Li T, Shan XJ, Zhang GH. Extremely Large Off-Fault Deformation during the 2021 Mw 7.4 Maduo, Tibetan Plateau, Earthquake. *Seismol. Res. Lett.* **94**, 39-51 (2023).

29. Provost F*, et al.* High-resolution co-seismic fault offsets of the 2023 Türkiye earthquake ruptures using satellite imagery. *Sci. Rep.* **14**, 6834 (2024).
