# Supplementary material for: Extensive off-fault damage around the 2023 Kahramanmaraş earthquake surface ruptures
Source: Nat Commun. 2025 Feb 3;16:1286. doi: 10.1038/s41467-025-56466-w (PMC11791046; doi:10.1038/s41467-025-56466-w)
Supplement: Supplementary file 2 — Description of Additional Supplementary Files [file 41467_2025_56466_MOESM2_ESM.pdf]

Description of Additional Supplementary Files

Supplementary Movie 1. The Supplementary Movie file contains fault-perpendicular profiles of fault-parallel displacements every 2 km along the main ruptures of the 2023 Kahramanmaraş (Türkiye) earthquakes. For each profile, the estimated total deformation, on-fault offset, absent surface displacement (ASD), ASD ratio, and damage width are labeled. Explanation of these parameters can be found in Fig. 2d of the main text. For profiles at 216-230 km of the first event, the observation significantly deviates from the arctan fitting, which is mainly due to the inelastic deformation around the Golbasl basin. Significant local horizontal and vertical deformation can be observed around this basin in the 3D displacement field (see Supplementary Fig. 1).
